# The Release of Grape Pomace Phenolics from Alginate-Based Microbeads during Simulated Digestion In Vitro: The Influence of Coatings and Drying Method

**DOI:** 10.3390/gels9110870

**Published:** 2023-11-01

**Authors:** Josipa Martinović, Jasmina Lukinac, Marko Jukić, Rita Ambrus, Mirela Planinić, Gordana Šelo, Gabriela Perković, Ana Bucić-Kojić

**Affiliations:** 1 Faculty of Food Technology Osijek, Josip Juraj Strossmayer University of Osijek, F. Kuhača 18, HR-31 000 Osijek, Croatia; josipa.grgic@ptfos.hr (J.M.); jasmina.lukinac@ptfos.hr (J.L.); marko.jukic@ptfos.hr (M.J.);; 2 Faculty of Pharmacy, Institute of Pharmaceutical Technology and Regulatory Affairs, University of Szeged, H-6720 Szeged, Hungary

**Keywords:** grape pomace, phenolics, encapsulation, drying techniques, microbeads characterization, release in vitro

## Abstract

Grape pomace is a byproduct of wineries and a sustainable source of bioactive phenolic compounds. Encapsulation of phenolics with a well-chosen coating may be a promising means of delivering them to the intestine, where they can then be absorbed and exert their health-promoting properties, including antioxidant, anti-inflammatory, anticancer, cardioprotective, and antimicrobial effects. Ionic gelation of grape pomace extract with natural coatings (sodium alginate and its combination with maltodextrins, gelatin, chitosan, gums Tragacanth and Arabic) was performed, and the resulting hydrogel microbeads were then air-, vacuum-, and freeze-dried to prevent spoilage. Freeze-drying showed advantages in preserving the geometrical parameters and morphology of the microbeads compared to other drying techniques. A good relationship was found between the physicochemical properties of the dried microbeads and the in vitro release of phenolics. Freeze-dried microbeads showed the highest cumulative release of phenols in the intestinal phase (23.65–43.27 mg_GAE_/g_MB_), while the most suitable release dynamics in vitro were observed for alginate-based microbeads in combination with gelatin, gum Arabic, and 1.5% (*w*/*v*) chitosan. The results highlight the importance of developing encapsulated formulations containing a natural source of bioactive compounds that can be used in various functional foods and pharmaceutical products.

## 1. Introduction

Grape pomace (GP) is a by-product of the wine industry that has attracted considerable attention due to its chemical composition (proteins, dietary fiber, pectin, oils, polyphenols, etc.), which is responsible for its potential health benefits, nutritional value, and functional properties [1,2,3,4,5,6]. The presence of polyphenols and other antioxidants in GP can help neutralize harmful free radicals in the body, thereby reducing oxidative stress and associated chronic diseases such as cardiovascular disease, cancer, and neurodegenerative disorders [7,8]. Moreover, GP has been found to have potential anti-inflammatory effects [3] and has also shown promise in the treatment of diabetes [8]. Dietary fiber, which is abundant in GP, is important for digestive health and preventing constipation [2], and contains various minerals and vitamins that are important for overall health and well-being [9].

In recent years, there has been an increased interest in the encapsulation of bioactive phenolic compounds from various natural sources [10] including GP. Encapsulation of phenolic compounds from GP using the ionic gelation method promises to significantly improve their stability, bioavailability, and controlled release. Ionic gelation is a method for encapsulating active compounds in crosslinked hydrogels formed by the interaction of oppositely charged ions, often using a polyanion and a cationic material [11]. This method is widely used due to its simplicity and ability to encapsulate a wide range of bioactive compounds, making it an important technique in pharmaceutical and biotechnological applications [12,13]. The hydrogels obtained after ionic gelation are susceptible to spoilage, therefore drying is an important step to ensure their long-term stability and preservation.

There is a constant search for new coatings and their combinations to obtain hydrogels based on natural polymers. The selection of specific coating materials has a significant impact on several critical hydrogel properties. These properties include geometrical parameters, textural attributes, morphological characteristics, swelling behavior, porosity, and a variety of other factors [11]. These elements, in turn, play a critical role in shaping the stability of the hydrogel microbeads and the controlled release of the encapsulated active ingredients. The choice of coatings essentially determines the structure and behavior of the hydrogel microbeads and influences how they interact with the encapsulated substances and the external environment, which ultimately affects the overall performance and functionality of the hydrogel system [11]. Each of the natural coatings used in this study–sodium alginate, maltodextrin, the gums Tragacanth and Arabic, gelatin from cold fish skin, and chitosan- has unique properties that may affect the encapsulation and release of phenolic compounds. Sodium alginate, a polysaccharide derived from brown algae, is most commonly used in various biomedical fields, including drug delivery [14], wound healing [15], tissue engineering [16], and regenerative medicine [17]. Due to its excellent properties such as biocompatibility, gel-forming ability, non-toxicity and biodegradability [14], sodium alginate is often used for ionic gelation of various compounds. On the other hand, maltodextrin is also commonly used due to its favorable properties including emulsification, water solubility, low viscosity at high concentrations, biodegradability, and film formation [18], but mainly for spray-drying and freeze-drying methods [19]. Maltodextrins have a dextrose equivalent (DE) that indicates the degree of hydrolysis or polymerization of starch molecules and affects its functional properties. Therefore, maltodextrins with lower DE values have longer glucose chains and higher molecular weights, impacting solubility, viscosity, and stability [20]. Gum Tragacanth and gum Arabic are both natural exudate gums that have been used as coatings for the encapsulation of various compounds [21]. Gum Arabic has been used for the encapsulation of grape seed oil [22], turmeric oleoresin [23], and blackberry derived bioactive compound [24], but it has also been shown to increase the stability of enzymes and probiotics [25,26]. Gum Tragacanth, although primarily studied for its rheological properties and structural components, also shows potential for antimicrobial and antioxidant applications, although the specific biomedical applications are less explored [27]. Fish gelatin has gained attention in various fields, including biomedical application and encapsulation. It offers several advantages over mammalian gelatin, such as economical production using discarded fish byproducts and fewer personal or religious restrictions [28]. Fish gelatin has been explored for its potential in wound dressing and healing, gene therapy, tissue engineering, implants, bone substitutes, and drug delivery systems [29]. Chitosan, a modified biopolymer derived from chitin, is used in various industries, including food, cosmetics, textile, and agriculture [30]. It has been used to encapsulate numerous compounds such as flavors, essential oils, vitamins, enzymes, and aroma to protect them from degradation and/or to control their release [30]. In addition to its encapsulation properties, chitosan coatings have shown antibacterial activity making them suitable for pharmaceutical and biomedical applications [31]. Chitosan has been used for wound dressing [32], drug delivery [33], and tissue engineering [34]. Overall, the choice of coating material in the encapsulation process has a significant impact on the encapsulation efficiency, physicochemical characteristics of the produced particles, and further use.

The objective of this study was to encapsulate Cabernet Sauvignon grape pomace extract (CSE) by the ionic gelation method using various alginate-based blends containing maltodextrins, Tragacanth and Arabic gums, cold fish skin gelatin, and chitosan. Subsequently, the effects of these blends and different drying techniques (air, vacuum and freeze-drying) on the size, shape, texture, morphology and in vitro release of phenolic compounds were investigated. The goal of this research was to improve the understanding of phenolic compound release, which has far-reaching implications for improving the controlled release, bioavailability, and targeted delivery of not only phenolic compounds but also other active ingredients in natural polymer-based systems.

## 2. Results and Discussion

### 2.1. Encapsulation Efficiency of the Total Phenolic Compounds

The efficiency of encapsulation (*EE*) of phenol-rich grape pomace extract (CSE) in various alginate-based blends was evaluated. This revealed the potential to improve *EE* by introducing various natural coatings in addition to sodium alginate.

**Figure 1:**
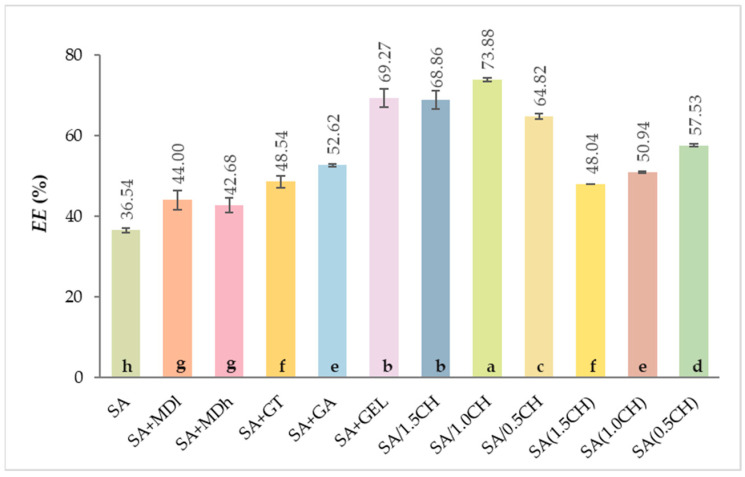
Encapsulation efficiency (*EE*, %) of grape pomace extracts using various coatings (SA—sodium alginate; MDl—maltodextrin DE 4–7; MDh—maltodextrin DE 16.5–19.5; GT—gum Tragacanth; GA—gum Arabica; GEL—gelatin; /CH—chitosan dispersed in crosslinking solution (0.5–1.5% *w*/*v*); (CH)—chitosan in immersing solution (0.5–1.5% *w*/*v*). Different letters represent significant differences (ANOVA, Duncan’s test at *p* < 0.05).

In Figure 1, the data illustrate that the use of sodium alginate (SA) alone resulted in the lowest *EE* of 36.54%.

When maltodextrins were combined with SA, the *EE* was statistically significantly increased (ANOVA, *p* < 0.05) and was 44.00% and 42.86% for maltodextrin with a lower dextrose equivalent, DE 4–7 (SA + MDl) and those with a higher dextrose equivalent, DE 16.5–19.5 (SA + MDh), respectively. However, the *EE* results, obtained with the two maltodextrins used were not statistically significantly different from each other, although the maltodextrin with a lower DE obtained a slightly better result. Maltodextrin with a lower DE indicates lower hydrolysis and larger molecules, which form an effective matrix for the encapsulation of phenolic compounds, as noted by Laine et al. [35] and also in combination with sodium alginate, as shown in in Figure 1.

The use of a mixture of SA with gum Tragacanth (SA + GT) and gum Arabic (SA + GA) further improved the *EE*, reaching 48.54% and 52.62%, respectively (Figure 1). It is known from the literature that the concentration and coating ratio play an important role in the encapsulation process. Apoorva et al. [36] investigated the influence of different ratios (100:0, 67:33, 50:50, 33:67) of 2% (*w*/*v*) SA and 2% (*w*/*v*) GT on the *EE* of phenolic compounds extracted from *Basella* species using the ionic gelation method. The *EE* values obtained ranged from 70% to 82%, and with an increase in alginate content, the *EE* increased proportionally. The *EE* value obtained with SA + GA was comparable to those found in the literature. In their study, Li et al. [37] encapsulated tea polyphenols with 2 wt% SA and 2 wt% GA at a ratio of 80:20, resulting in an *EE* of 48.56%. In addition to the concentration used, the structure of the coatings also plays a role in the encapsulation process. GT is mainly composed of branched polysaccharides, while GA is a complex mixture of polysaccharides, glycoproteins, and other compounds with a highly branched structure [38]. The structure of GA and its functional groups promote favorable interactions with phenolic compounds [39] and retain them in the hydrogel. Similar interactions with phenolic compounds are formed by proteins such as gelatin (GEL) through various types of bonds, including hydrogen bonding, electrostatic interactions, and hydrophobic interactions [40], which improves *EE*. As shown in Figure 1, the addition of GEL to SA (SA + GEL) improved the *EE* (69.27%).

When chitosan (CH) was combined with SA and dispersed in the crosslinking solution CaCl_2_ at three concentrations (1.5%-SA/1.5CH, 1.0%-SA/1.0CH, and 0.5%-SA/0.5CH), the percentage of *EE* ranged from 64.82% to 73.88%, with SA/1.0CH having the highest *EE* compared to all prepared mixtures (Figure 1). In this way, the chitosan is immediately incorporated into the hydrogel matrix during encapsulation and can therefore be more uniformly distributed in the hydrogel. When alginate hydrogels were immersed in CH at three concentrations (1.5%-SA(1.5CH), 1.0%-SA(1.0CH), 0.5%-SA(0.5CH)), the *EE* was lower than the previous case and ranged from 48.04% to 57.53% (Figure 1). At high concentrations, the chitosan molecules in solution may start to aggregate or precipitate, which in return leads to a lower *EE* [41]. Overall, chitosan has a positive charge due to the amino groups, while phenolic compounds often have a negative charge, and this electrostatic attraction can improve *EE* [42].

### 2.2. Hydrogel Microbeads and Dried Microbeads Characterization

#### 2.2.1. Size, Shape, and Morphology

After CSE encapsulation, the hydrogel microbeads were stabilized by three drying techniques: air drying, vacuum drying, and freeze-drying to prevent their spoilage. The hydrogel microbeads and the dried microbeads were characterized by their geometrical properties, especially the size and shape parameters, while the morphology of the dry microbeads was evaluated by scanning electron microscope (SEM), since the above parameters are important for understanding the release of the active ingredient from the microbeads into the digestive tract. The effects of drying method and coating on the ability to release phenolic compounds from the dried microbeads in digestive juices were studied in vitro.

The results of the analysis of the size and shape parameters presented in the attached materials (Tables S1–S7) show that the coatings used have an influence on the studied size and shape parameters of the hydrogel microbeads. For the dried microbeads, the size and shape parameters were influenced by the coatings used and the drying process individually or in interaction, as each drying techniques in combination with the individual coating(s) had a different influence on the size and shape parameters studied.

Considering only the hydrogel microbeads, it can be seen that the smallest microbeads were obtained with only one coating for CSE encapsulation, i.e., SA, where the average surface area of a particle was 11.84 mm^2^ (Table S1), the perimeter was 13.21 mm (Table S2), and the maximum and minimum Feret were 4.24 mm and 3.65 mm, respectively (Tables S3 and S4). The addition of the subsequent coating to SA affected the enlargement of the hydrogel microbeads, with the largest microbeads formed when the SA/1.5CH coating was used. These particles had a projected area of 19.32 mm^2^ (Table S1), perimeter of 17.91 mm (Table S2), a maximum Feret of 6.65 mm (Table S3), and a minimum Feret of 3.89 mm (Table S4).

As expected, drying removed some of the moisture from the hydrogel microbeads, resulting in a significant reduction in size for all dried microbeads. In general, the microbeads showed the least changes in size parameters after freeze-drying, while the largest changes were observed in the air-dried microbeads compared to the hydrogel microbeads. More specifically, the area reduction (Table S1) of the microbeads after freeze-drying compared to the hydrogel microbeads ranged from 31% (SA + GA) to 62% (SA), which was statistically less significant (*p* < 0.05) than the area reduction after vacuum drying, which ranged from 80% (SA + MDl, SA + GEL) to 88% (SA(0.5CH)), and after air drying, where the area reduction ranged from 83% (SA + MDl, SA + GEL) to 89% (SA(1.5CH), SA(1.0CH)). A similar trend is observed in the reduction of the perimeter of the dried microbeads compared to the hydrogel microbeads (Table S2), where the smallest change in the perimeter of the microbeads after freeze-drying ranged from 7% (SA + GA) to 35% (SA), more significant changes in the perimeter were then observed after vacuum drying, ranging from 53% (SA + MDl) to 67% (SA(0.5CH)), and the largest decrease in perimeter was observed after air drying, ranging from 59% (SA + GT) to 67% (SA(1.5CH), SA(1.0CH)). The Feret_MAX_ values presented in Table S3 show that the highest value was attributed to the dried microbeads SA/1.5CH, regardless of the drying technique used. After the drying process, the SA + GEL microbeads consistently had the highest Feret_MIN_ values for all drying techniques (Table S4).

As mentioned above, the drying process and the coating(s) used, as well as their interactions, had a statistically significant (*p* < 0.05) influence on the shape parameters, circularity, roundness, and solidity, of the dried microbeads, while the coating used influenced the tested shape parameters of the hydrogel microbeads. Table S5 shows the circularity results for the hydrogel microbeads and the dry microbeads, quantifying the degree of conformity to a perfect circle, with values ranging from 0 to 1, where 1 represents a perfect circular shape. Hydrogel and air-dried microbeads had the least deviation from the regular shape of a circle, with a range of values for circularity of 0.76 (SA/1.5CH) to 0.88 (SA(1.0CH), SA(0.5CH)) for hydrogel microbeads and for air-dried microbeads from 0.74 (SA/1.5CH, SA/0.5CH) to 0.89 (SA(0.5CH)). Vacuum-dried microbeads had a lower value for circularity (0.68–0.89), while freeze-dried microbeads had the largest deviation from the shape of a circle with circularity ranging from 0.63 (SA/1.5CH) to 0.78 (SA + MDl).

The roundness quantifying the curvature of the edges and corners of hydrogel microbeads and dried microbeads, is detailed in Table S6. For all three drying techniques, the roundness of the dried microbeads generally decreased compared to the hydrogel microbeads. The roundness of the hydrogel microbeads ranged from 0.59 to 0.91, and that of the air-dried microbeads ranged from 0.56 to 0.88, while the greatest deviation in roundness values from the hydrogels was observed for the microbeads obtained by vacuum drying and freeze-drying, which ranged from 0.52 to 0.89 (Table S6).

Table S7 presents the solidity values of the examined samples. Solidity is a shape parameter that serves as an indicator of the compactness and smoothness of the particle, with values falling within the range of 0 to 1, where 1 signifies a highly compact particle. For the hydrogel microbeads, the solidity values ranged from 0.97 to 0.98 (Table S7). However, after the drying process, these values decreased, indicating a deviation from the regular shapes and the presence of voids in the samples. Nevertheless, after drying, the solidity values remained in the range of 0.92 to 0.97, but there was a statistically significant difference (*p* < 0.05) compared to the hydrogels, with a greater deviation observed in the vacuum- dried and freeze-dried microbeads than in the air-dried microbeads (Table S7). It is important to note that solidity refers exclusively to the edges and borders of both the hydrogel microbeads and the dried microbeads, so a more detailed study of microbead morphology was performed using SEM.

Considering all the shape parameters determined (Tables S5–S7), it can be generally concluded that the microbeads (hydrogel and dried microbeads) exhibited the most irregular shape when CSE was encapsulated with combination of SA and CH dispersed in crosslinking solution, and the irregularity of the particles increasing with increasing CH concentration.

The morphology of the dried microbeads investigated by SEM is shown in Supplementary Materials (Figure S1), whereas morphology of the microbeads (SA + GT), (SA/1.5CH), and (SA(1.5CH)–SA(0.5CH)) are shown in following paragraphs.

**Figure 2:**
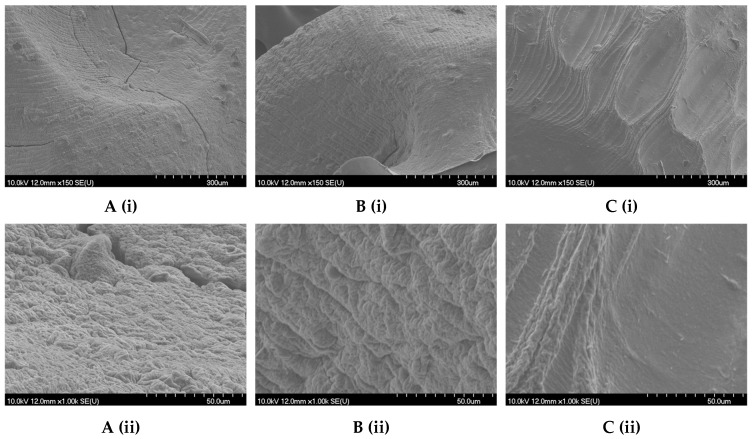
SEM image of (**A**) air-dried microbeads, (**B**) vacuum-dried microbeads and (**C**) freeze-dried microbead of grape pomace extract encapsulated with a combination of sodium alginate and Tragacanth gum as a coating (SA + GT); and their outer layer with the scale bar of 300 µm (**i**) and 50 µm (**ii**).

Air-dried microbeads exhibited surface furrows and cracks (Figure 2A), as in the study published by Saarai et al. [43], although these features were less pronounced after vacuum drying (Figure 2B), similar to the results of Keskin et al. [44] and Anbinder et al. [45]. Surface depressions were observed in the freeze-dried microbeads, as in other articles [46,47], but no cracks were visible at higher magnification (Figure 2C).

**Figure 3:**
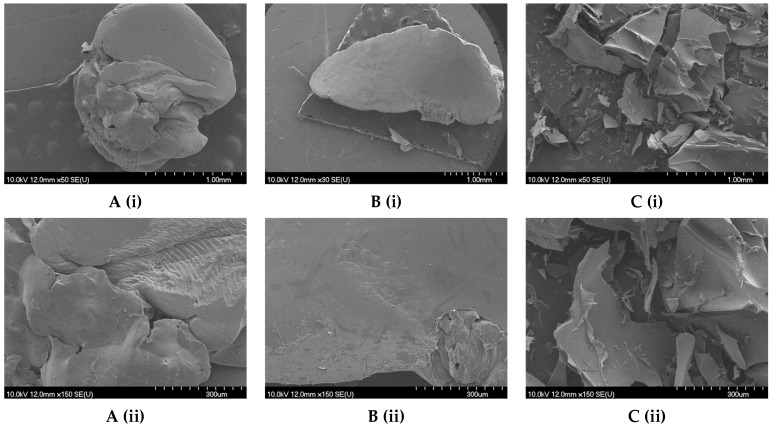
SEM image of (**A**) air-dried microbeads, (**B**) vacuum-dried microbeads and (**C**) freeze-dried microbead of grape pomace extract encapsulated with a combination of sodium alginate and 1.5% (*w*/*v*) chitosan dispersed in calcium chloride (SA/1.5CH); and their outer layer with the scale bar of 1 mm (**i**) and 300 µm (**ii**).

Air drying often leads to uneven drying, resulting in the formation of cracks, deformations, or inconsistencies in particle size and shape, which can be troublesome when uniformity is crucial. Hydrogels are also prone to shrinkage and distortion during air drying due to the gradual evaporation of water, which typically starts at the outer layers and progresses inward [48]. The SEM images offer insights into the morphology of the SA/1.5CH microbeads and reveal their distinct characteristics. Notably, the air-dried microbeads exhibit a deformed structure, as evidenced in Figure 3A, while those subjected to vacuum drying display a prominent ellipsoidal shape, as evident in Figure 3B. These observations corroborate the circularity and roundness parameters measured (Tables S5 and S6). Furthermore, air- and vacuum-dried microbeads are seen whole on the SEM images at the same magnification and size scale, while freeze-dried ones are not (Figure 3C). This confirms the differences in size parameters area and perimeter found for SA/1.5CH microbeads (Tables S1 and S2). A similar case was noticed for SA/1.0CH microbeads, where 1.0% (*w*/*v*) chitosan was dispersed in CaCl_2_ (see Supplementary Materials Figure S1).

**Figure 4:**
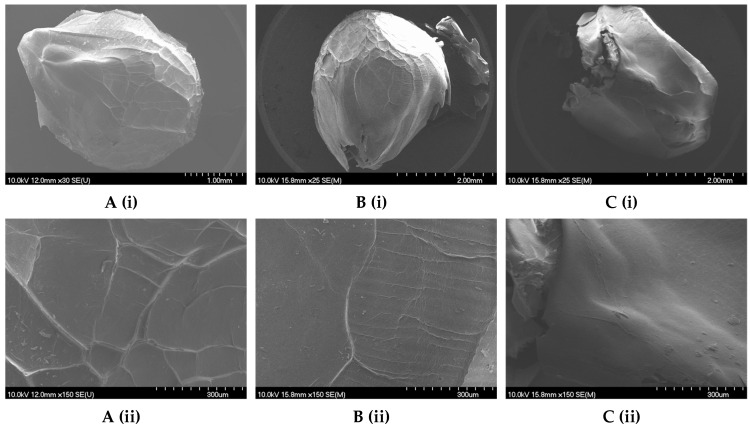
SEM image of freeze-dried microbead of grape pomace extract encapsulated with a combination of sodium alginate and (**A**)—1.5% (*w*/*v*) chitosan (SA(1.5CH)), (**B**)—1.0% (*w*/*v*) chitosan (SA(1.5CH)), and (**C**) 0.5% (*w*/*v*) chitosan (SA(0.5CH)) in immersion solution; and their outer layer with the scale bar of 1 mm and 2 mm (**i**) and 300 µm (**ii**).

Freeze-dried SA(1.5CH) and SA(1.0CH) microbeads exhibited a smooth surface with minimal folds and a distinct teardrop-like shape (Figure 4A,B), while SA(0.5CH) microbeads did not exhibit a similar shape but retained a smooth surface (Figure 4C).

Figure 3 and Figure 4 illustrate the significant influence of the use of identical coatings on the microbead morphology and geometric characteristics, while emphasizing the impact of CH concentration as well. Additionally, freeze-drying has notable advantages for maintaining geometric parameters and morphology of microbeads compared to air and vacuum drying, because water is sublimated slowly from the frozen state [49,50]. While the dried microbeads retain their initial contours, it is worth noting that surface imperfections such as dents, may arise [48] as seen in the SEM images displayed in Supplementary Materials (Figure S1). Nonetheless, this conservation of the original structure allows freeze-dried microbeads to closely mimic the characteristics of hydrogel microbeads.

#### 2.2.2. Texture

The texture parameter, hardness, was evaluated for both the hydrogel microbeads and the dried microbeads, and the results are shown in Table S8.

The results show that different coatings and drying techniques have an impact on the hardness of the hydrogel microbeads. Bušić et al. [51] performed encapsulation of dandelion polyphenols in alginate hydrogels coupled with whey protein isolate, cocoa powder, or carob as additional coatings, and their results showed increased strength compared to pure alginate hydrogels. In this study, the SA + MDh hydrogel microbeads had the highest measured hardness (0.44 N), but it was not statistically significantly different from SA + GEL, SA/1.5CH, SA/0.5CH, and SA(0.5CH).

In addition, air drying and vacuum drying significantly increased the hardness of the microbeads compared to the hydrogel microbeads, as shown in the results presented in Table S8. Hydrogel microbeads can retain large amounts of liquid due to their hydrophilicity [52]. Therefore, the increase in hardness can be attributed to the removal of water content during the drying process, resulting in the dried microbeads having a more compact structure that requires greater force for compression.

When comparing the dried microbeads, a different effect of drying method on hardness was found. Vacuum drying resulted in greater changes in microbead hardness, with SA(0.5CH) microbeads having the highest value. On the other hand, freeze-drying had a lesser effect on hardness compared to the hydrogel microbeads, with the SA microbeads having the highest hardness of 3.40 N.

### 2.3. In Vitro Release of Encapsulated Phenolic Compounds

Considering that the potential site of absorption of phenolic compounds is the intestinal phase, the increase of total phenolic compounds (TPC) release in the intestinal phase (IP) was a more important criterion for the selection of a suitable coating and drying method of hydrogel microbeads. Results obtained in this study showed that the coatings and drying methods used affect the release of TPC from the dried microbeads.

The concentration profile of the cumulative release of TPC from air-dried, vacuum-dried, and freeze-dried microbeads prepared with 12 coatings was evaluated for the oral (OP), gastric (GP), and IP phases of simulation digestion in vitro without digestive enzymes (Figures S2–S5). In general, a similar trend of increasing TPC release was observed through three digestion phases, regardless of the drying technique of the hydrogel microbeads although the highest cumulative release was observed in IP during for freeze-dried (23.65–43.27 mg_GAE_/g_MB_), followed by vacuum-dried (23.51–35.41 mg_GAE_/g_MB_) and finally air-dried (22.74–31.38 mg_GAE_/g_MB_) microbeads.

Freeze-drying is a widely used method to preserve phenolic compounds after ionic gelation because the structure of the microbeads is preserved (Figure S1) and the surface area is increased by removing ice crystals, creating dents that promote faster diffusion of the phenolic compounds. Abdin et al. [53] found similar results, with higher release of TPC from freeze-dried microbeads compared to vacuum-dried ones. Air drying exposes the hydrogel microbeads to humidity, light, and temperature fluctuations, which can lead to their decomposition and thus unstable and unpredictable release (Figures S2–S5). Air-dried microbeads were the smallest (Tables S1–S4) and had the highest hardness (Table S8). Their dense structure hinders the penetration of digestive fluids, which reduces the release of phenolic compounds, similar to the results published by Guzman-Villanueva et al. [54]. In addition, air-dried microbeads showed the lowest deviation from sphericity (Tables S5 and S6) (Figure S1), implying that the contact area with digestive fluids is smaller, resulting in a lower concentration of released TPC. Vacuum drying is gentler than air drying and has a favorable effect on the stability of phenolic compounds [55]. The advantage over air drying is the controlled drying conditions, which result in a product of more uniform quality. Compared to air-dried microbeads, vacuum-dried microbeads had slightly larger size parameters (Tables S1–S4), higher deviation from the circle (Tables S5 and S6) (Figure S1), and lower hardness (Table S8), resulting in a similar release pattern but with a slightly higher content of released TPC (Figures S2–S5).

It was also found that the coatings used had different effects on the release of phenolic compounds, and the highest cumulative content of TPC in the IP was observed when using CH dispersed in CaCl_2_ (40.78–43.27 mg_GAE_/g_MB_), followed by CH, when it was used as an immersion solution for hydrogel microbeads (35.47–36.86 mg_GAE_/g_MB_), and then GT (31.02 mg_GAE_/g_MB_), GA (29.77 mg_GAE_/g_MB_), GEL (28.05 mg_GAE_/g_MB_), SA (27.77 mg_GAE_/g_MB_), MDh (24.50 mg_GAE_/g_MB_), and MDl (23.65 mg_GAE_/g_MB_). The results show that SA, as one of the most commonly used coatings to protect bioactive substances, has a lower cumulative content in IP compared to most of the tested coatings, except in combination with maltodextrins. Bušić et al. [51] found that SA microbeads have a porous structure and tend to release their contents quickly, and it is also visible in this study in Figure S1. Furthermore, since SA microbeads are porous and not stable enough in the low pH gastric environment the encapsulated compounds are faster released [56], and so there is justification for applying an additional coating to improve effect a more controlled release of TP and its delivery into the IP.

**Figure 5:**
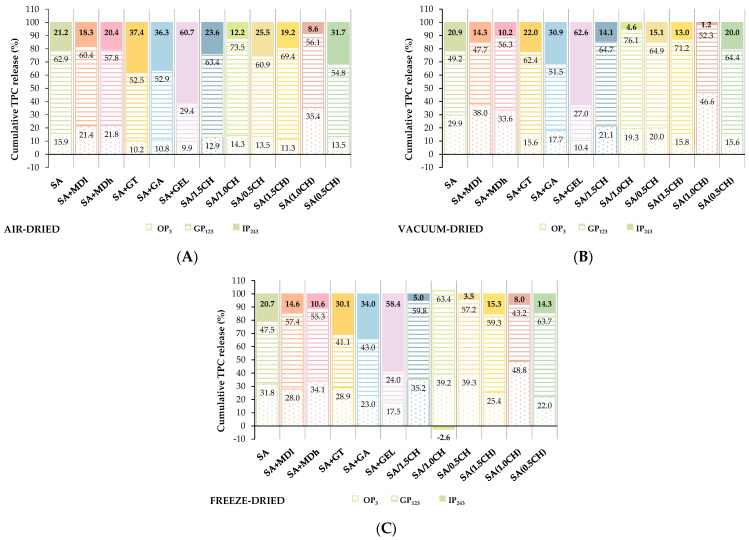
The percentage of cumulative TPC release for each gastrointestinal phase: OP-oral phase, GP-gastric phase, IP-intestinal phase for air-dried (**A**), vacuum-dried (**B**) and freeze-dried (**C**) microbeads (SA—sodium alginate; MDl—maltodextrin DE 4–7; MDh—maltodextrin DE 16.5–19.5; GT—gum Tragacanth; GA—gum Arabica; GEL—gelatin; /CH—chitosan dispersed in crosslinking solution (0.5–1.5% *w*/*v*); (CH)—chitosan in immersing solution (0.5–1.5% *w*/*v*; mage of the freeze-dried microbeads are included in the manuscript).

The percentage of cumulative TPC release in the three digestion phases for the tested coatings is shown in Figure 5A–C.

These data indicate the percentage of TPC released in each phase relative to the total percentage of TPC released during the 243-min digestion. The highest cumulative release of TPC (58.4–62.6%) during the IP and lowest value during GP (24.0–29.4%) and OP (9.9–17.5%) (Figure 5A–C), which is the most desirable release profile (Figure S3E), was observed for SA + GEL microbeads, regardless of the drying technique used. This could be related to the fact that the microbeads swell better at intestinal pH, which allows lower diffusion of TPC in GP and higher in IP, as found by Devi et al. [57].

Moreover, SA + GT and SA + GA released a similar percentage of TPC in IP: 22.0–37.4% and 30.9–36.3%, respectively. Their better TPC release compared to SA at intestinal pH may be attributed to the electrostatic repulsion between the negatively charged carboxyl groups in sodium alginate, gum Arabic and gum Tragacanth, which promotes greater swelling of the microbeads [36,58]. Tsai et al. [59] also observed increased release of phenolic compounds in IP when they encapsulated radish by-product juice with an SA + GA coating combination compared to SA. Similarly, Apoorva et al. [36] reported an increased release of phenolic compounds extracted from *Basella* species when a combination of SA + GT was used.

Generally, when chitosan was applied, it was found that SA/1.0CH and SA(1.0CH) microbeads exhibited the least desirable release dynamics regardless of the drying technique (Figure 5A–C). Figure 5C even shows a negative cumulative percentage (−2.6%) of TPC release in the IP phase for SA(1.0CH) microbeads. On the other hand, considering that freeze-drying was shown to be the best method in terms of TPC release (Figures S2–S5), it can be concluded that among all tested microbeads prepared with chitosan, the highest percentage of TPC was released in the IP from SA(1.5 CH) microbeads (15.3%) (Figure 5C). A similar conclusion was reached by Dai et al. [60] when they studied the effects of these two methods of addition chitosan on the release of nifedipine. They observed a better release profile of microbeads immersed in a 0.1% (*w*/*v*) chitosan solution than when chitosan was dispersed in a CaCl_2_ solution, because the microbeads swell more in the higher pH range due to less bonding between the carboxyl groups of alginate and the amino groups of chitosan [60].

When chitosan is added to the crosslinking solution and becomes an integral part of the hydrogel network, it can interact with phenolic compounds via hydrogen bonding and electrostatic interactions [61]. However, extensive optimization may be required to obtain a specific release profile, as the distribution of chitosan in the hydrogels may not be uniform and may lead to variations in the release rate. On the other hand, it the hydrogel microbeads are immersed in chitosan after formation, the primary interaction between chitosan and hydrogel occurs at the surface, resulting in a lower concentration of chitosan in the bulk of the hydrogel [62]. Sustained release of phenolic compounds from the bulk of the microbeads tends to occur more slowly because of the limited penetration of chitosan into the deeper layers of the hydrogel matrix [45,63]. Belščak-Cvitanović at al. [64,65] also observed comparable outcomes, where chitosan coating prolonged the release of green tea phenolic compounds and caffeine from alginate microbeads.

The percentage of cumulative release in IP of MDl microbeads (14.3–18.3%) and MDh microbeads (10.2–20.4%) was lower than that of SA microbeads (20.7–21.2%) regardless of the drying method (Figure 5A–C). It can be assumed that due to a higher percentage of TPC release in OP from MD (21.4–38.0% for MDl and 21.8–34.1% for MDh) than from SA microbeads (15.9–31.8%) there is a decrease in the cumulative TPC content in IP (Figure S3A,B). Although the results of this study indicate a similar pattern of TPC release for both tested MD, generally MDh contain more reducing sugar groups, thereby expediting phenolic compound release. MDh are more likely to form stable complexes with phenolic compounds, but the formed hydrogels degrade faster, enabling rapid release. Maltodextrins, generally, exhibit excellent chemical stability across a wide pH rang, rendering them suitable for various food and pharmaceutical applications [66].

In the overall view of all the results of this comprehensive investigation, SA + GEL, SA + GA, SA and SA(1.5CH) were found to be the best coatings in terms of release of phenolic compounds in the IP phase. The aforementioned coatings were further used to describe the release kinetics with mathematical models and to evaluate the bioaccessibility of the phenolic compounds, and the results were published in two scientific papers [67,68].

## 3. Conclusions

This study demonstrates the crucial role of the drying technique and the coatings used on the properties of the microbeads containing phenol-rich grape pomace extract and consequently on phenolics bioaccessibility, which has valuable implications for further applications in the food and pharmaceutical industries.

The results show that the tested coatings for the encapsulation of grape pomace extract in hydrogel microbeads significantly affect the encapsulation efficiency (*EE*) and their geometrical parameters. The addition of a second coating to sodium alginate (SA) resulted in an increase in *EE* by 1.2–2.0 times compared to SA and an increase in the surface area of hydrogel microbeads by 1.1–1.6 times. The highest *EE* of 73.88% was achieved when 1.0% (*w*/*v*) chitosan was used as second coating and dispersed in the crosslinking solution (SA/1.0CH), and the microbeads prepared in the same way but with a concentration of 1.5% (*w*/*v*) chitosan (SA/1.5CH) had the largest surface area (19.32 mm^2^).

The coatings and drying technique used (air, vacuum, and freeze drying) also had a significant effect on the size, shape, and texture of the dried microbeads. Freeze-drying caused the least reduction in size (between 31% and 62% compared to the original hydrogel microbeads) and the least change in morphology. Vacuum and air drying significantly increased the hardness of the microbeads compared to freeze-dried microbeads due to the different mass transfer between them, which affected their more compact structure compared to the porous structure of the freeze-dried microbeads.

The importance of the encapsulation process also extends to the release of phenolic compounds during simulated in vitro digestion. Freeze-drying proved to be the most effective technique for preserving the structure of the microbeads and creating favorable conditions for rapid diffusion of the phenolic compounds. This was reflected in the highest cumulative release observed in the intestinal phase (IP), ranging from 23.65 to 43.27 mg_GAE_/g_MB_ for freeze-dried microbeads, followed by vacuum-dried (23.51–35.41 mg_GAE_/g_MB_) and air-dried (22.74–31.38 mg_GAE_/g_MB_) microbeads. The choice of coating also had a significant effect on the release of phenolic compounds, with freeze-dried microbeads coated with chitosan dispersed in CaCl_2_ having the highest cumulative content in IP (40.78–43.27 mg_GAE_/g_MB_). Coatings such as SA exhibited lower cumulative content of phenolics in IP, highlighting the need for additional coatings for controlled release and better bioaccessibility in IP. The SA + gelatin coating had the best release profiles (58.4–62.6% of phenolic compounds released in IP of total phenolics released during in vitro digestion), followed by the SA + gum Arabic, and SA + 1.5% *w*/*v* chitosan (used as an immersion solution) coatings. This result underlines their potential to significantly improve bioaccessibility in IP.

Overall, grape pomace is a valuable resource for natural phenolic antioxidants that can contribute to health maintenance. Their effect depends on the formulation introduced into the body and bioaccessibility in the small intestine, which is a crucial site for the absorption of phenolic compounds and the unfolding of their potential biological activity and health-promoting effects. With this in mind, future research should focus on developing new mathematical models to clarify and predict the release kinetics of total phenolic compounds from the prepared formulation during simulated in vitro digestion. In addition, the possibility of using encapsulated phenol-rich grape pomace extracts in the formulation of various food products (e.g., biscuits) will be investigated and the bioaccessibility of the phenolic compounds after digestion will be evaluated. In addition, clinical studies with an interdisciplinary approach are needed to confirm the hypothesis on the health benefits of polyphenols from grape pomaces. In the meantime, we can do a lot for our health with small steps by including encapsulated grape pomace extract in our daily diet, e.g., by incorporating them into breakfast (smoothies, muesli, yogurt).

## 4. Materials and Methods

### 4.1. Reagents and Chemicals

The coatings used were alginic acid sodium salt (SA) from brown algae (low viscosity), gelatin (GEL) from cold water fish skin, gum Arabic (powder) (GA), medium molecular weight chitosan (deacetylated chitin) (CH), gum Tragacanth (GT), maltodextrin (DE 4–7) (MDl), maltodextrin (DE 16.5–19.5) (MDh), and standard gallic acid monohydrate (98+% A. C. S. reagent) were purchased from Sigma Aldrich (Saint Louis, MO, USA). Folin and Ciocalteu’s phenol reagent was purchased from CPAchem (Bogomilovo, Bulgaria), 96% ethanol (p.a.) from Lab Expert (Shenzhen, Guangdong, China), and glacial acetic acid (99.5%) was purchased from Macron Fine Chemicals (Gliwice, Poland). The salts used to prepare the electrolyte solutions were purchased from Acros Organics (Geel, Belgium), Gram Mol (Zagreb, Croatia), and Kemika (Zagreb, Croatia).

### 4.2. Grape Pomace

Pomace of Cabernet Sauvignon (*Vitis vinifera* L.) variety grape (CS) left after vinification was obtained from Erdut winery (Erdut, Croatia, 2018 harvest). It consisted of grape skins, seeds, and pulp. The pomace was air dried (48 h, 25–27 °C) to reduce the moisture content from 48.60% to 8.41% and stored at room temperature. Before use, the grape pomace was ground to a particle size of up to 1 mm using an ultracentrifugal mill (Retsch ZM200, Haan, Germany).

### 4.3. Phenol-Rich Grape Pomace Extract Preparation

Phenolic compound extraction from CS was performed in a shaking water bath (Julabo, SW-23, Seelbach, Germany) at 80 °C and 200 rpm for 120 min, using a 50% aqueous ethanol solution as solvent with a grape pomace-solvent ratio of 40 mL/g. After extraction, samples were centrifuged at 11,000× *g* for 10 min (Z 326 K, Hermle Labortechnik GmbH, Wehingen, Germany). The resulting liquid phenol-rich extract was concentrated to dryness in rotary evaporator at 48 mbar and 50 °C (Büchi, R-210, Flawil, Germany) and that dry extract (CSE) was used for encapsulation.

### 4.4. Determination of Total Phenolic Content

Total phenolic content (TPC) was determined using the Folin–Ciocalteu method described by Waterhouse [69], with some modifications. Briefly, 3160 µL of distilled water was mixed with 40 µL of sample and 200 µL of Folin–Ciocalteu reagent. After an 8-min incubation period, 600 µL of 20% (*w*/*v*) sodium carbonate was added, and the mixture was further incubated at 40 °C. After 30 min absorbance was measured at 765 nm against a blank containing distilled water instead of the sample. The results were expressed as gallic acid equivalents (GAE) per g of sample used (extract or microbeads).

### 4.5. Encapsulation by Ionic Gelation

The prepared CSE (1.0 g) was dissolved in 30% aqueous ethanol solution (20.8 mL) and distilled water (79.2 mL) through continuous mixing on a magnetic stirrer for 180 min. To eliminate any undissolved extract particles, the mixture underwent centrifugation at 15,000× *g* for 5 min, after which clear supernatant (90 mL) was separated and used for encapsulation [67]. The rest of the supernatant was used for the determination of TPC.

Twelve different types of hydrogel microbeads were prepared by ionic gelation with a total of 7 different coatings (Table 1).

In all 12 types of hydrogel microbeads, SA was used as the primary coating, whereas the secondary coating (MDl, MDh, GT, GA, GEL or CH) was added to the encapsulation liquid (experimental set #: 2–6), i.e., in crosslinking solution (experimental set #: 7–9) or immersion solution (experimental set #: 10–12).

Ionic gelation was performed using a Büchi B-390 encapsulator (Flawil, Switzerland). A 450 µm diameter nozzle was operated at a frequency of 140 Hz and an electrode voltage of 750 V was used during the experiments. The working pressure is set as a function of the viscosity of the encapsulation feed (mixture of coating and active ingredient). To achieve homogeneity of the encapsulation feed and improve the adhesion of the phenolic compounds to the coating(s), the mixture was continuously stirred for 24 h prior to encapsulation. The prolonged mixing also served to remove air bubbles that could interfere with the encapsulation process. Ionic gelation was performed dropwise in 300 mL of crosslinking solution (0.25 M CaCl_2_), which facilitated the formation of hydrogel microbeads. The hydrogel microbeads were then mixed gently mixed in the crosslinking solution for 10 min to ensure complete solidification. The hydrogel microbeads were then washed twice with 200 mL of distilled water to remove residual non-crosslinked calcium ions adhering to the hydrogel surface [67].

CH as a secondary coating was added using 2 different techniques to prepare hydrogel microbeads. In the first technique, CH was dispersed in a CaCl_2_ solution 24 h before encapsulation to ensure complete dissolution. CH and 0.25 M CaCl_2_ were dissolved in a 1% glacial acetic acid solution at the concentrations shown in Table 1. Another procedure consisted of preparing an alginate hydrogel microbeads that was immediately immersed in various CH solutions (Table 1) after solidification in a CaCl_2_ solution for 10 min. These hydrogel microbeads were mixed in the appropriate CH solutions for 10 min and then washed twice with 200 mL distilled water. The CH solutions were prepared 24 h before encapsulation with 1% glacial acetic acid solution to ensure complete dissolution.

### 4.6. Encapsulation Efficiency Determination

After completion of hydrogel preparation, the encapsulation efficiency (*EE*, %) was calculated from the TPC released into the crosslinking solution and washed off the surface of the hydrogels and the initial TPC in the CSE used for encapsulation according to the following Equation (1):

(1) $$EE\;\left(\%\right)=\frac{C_{E}-C_{W}}{C_{E}}\;\cdot 100$$

where *C_E_* is the initial TPC (mg), measured in the supernatant of the phenol-rich supernatant prepared for encapsulation and *C_W_* is the TPC (mg) found in the combined calcium chloride and wash water as well as chitosan when used. The results obtained are given as the mean of replicates ± SD.

### 4.7. Hydrogel Microbeads Drying Techniques

Hydrogel microbeads prepared by ionic gelation are unstable and susceptible to contamination and spoilage due to high water content. For this purpose, the drying of hydrogels has been carried out by the following three techniques: air drying, vacuum drying, or freeze-drying. In air drying, the hydrogel microbeads were placed on a glass Petri dish so that they did not touch each other and dried at room temperature for 24 h. In vacuum drying, the hydrogel microbeads were placed in Petri dishes so that they did not touch each other and dried in a preheated vacuum dryer (WOV-30, Witeg, Wertheim, Germany) at 50 °C and a pressure of 300 mbar for 5 to 8 h, depending on the sample. In freeze-drying [67], hydrogel microbeads previously frozen at −80 °C (SWUF Ultra Low Temperature Smart Freezer, Witeg, Wertheim, Germany) were dried in a freeze-dryer (Freeze-dryer Alpha 2–4 LSCplus, Christ, Osterode am Harz, Germany) at −83 °C under a vacuum of 0.250 mbar for 24 to 72 h, depending on the sample.

### 4.8. Characterization of Hydrogel and Dried Microbeads

#### 4.8.1. Determination of Geometric Characteristics

Computer image analysis was used to investigate the geometric characteristics of size and shape for both hydrogels and dried microbeads. The EPSON V500 Photo Scanner (Epson America Inc., Long Beach, CA, USA), operating at 800 dpi resolution and 24-bit color depth in the sRGB model, was used to capture and digitize samples in TIFF format. Each set of experiments consisted of exactly 10 hydrogels or dry microbeads carefully arranged on a glass Petri dish to avoid contact. The Petri dish containing the samples was then placed in a dark chamber to exclude external light and minimize scanning errors. After scanning, the acquired images were thoroughly postprocessed using the ImageJ program (version 1.59g, Wayne Rasband, NIMH, Bethesda, MD, USA). The observed parameters were divided into two groups: size-related parameters included area, perimeter, and maximum and minimum Feret diameters, while shape-related parameters included circularity, roundness, and solidity. To ensure accurate measurement, the results obtained for the size parameters of the samples were converted from pixel units to millimeters, considering the known values of the resolution of the scanner’ in dpi units. Shape parameters were calculated using ImageJ User Guide-IJ 1.46r software [70]. All measurements were performed in triplicate and are given as mean values of the measurements ± SD.

#### 4.8.2. Scanning Electron Microscopy (SEM)

The morphology of dried microbeads was examined using scanning electron microscopy (Hitachi S4700, Hitachi Scientific Ltd., Tokyo, Japan) at 10 kV. Prior to imaging, a thin gold-palladium film was applied to the dry microbeads using a sputter coater (Bio-Rad SC 502, VG Microtech, Uckfield, UK).

#### 4.8.3. Texture Determination

The texture profile of hydrogels was evaluated using the TA.XTplus Texture Analyzer (Stable Microsystems Ltd., Surrey, UK). Each individual hydrogel underwent a dual compression cycle of 50%, with a 2 s interval between compressions and a test speed of 0.5 mm/s. An aluminum cylinder probe with a diameter of 10 mm was used for this purpose. The hardness value derived from the texture profile analysis curves, was determined after a 2 s compression when the hydrogel was compressed to 1 mm. A similar procedure was used for the dried microbeads, using the same probe to perform compression with a load of 20% at a test speed of 0.1 mm/s and determining the hardness at the maximum peak height. The texture profile of ten individual hydrogels and dried microbeads of each sample was evaluated and the hardness was reported as the mean of the measurements ± SD.

### 4.9. Release Study of Encapsulated Phenolic Compounds from Grape Pomace Extract

The in vitro release of total phenolic compounds from the air, vacuum, and freeze-dried microbeads was conducted following the INFOGEST protocol [71], with some modifications. The release study included oral phase (OP), gastric phase (GP), and intestinal phase (IP), simulating the conditions of the human gastrointestinal tract. Electrolyte solutions mimicking these phases were utilized, and the process was enzyme-free. Throughout the entire 243-min experiment, the temperature was maintained at 37 °C, and stirring was carried out using a magnetic stirrer.

To initiate the OP, 100 mg of microbeads were combined with 4 mL of simulated salivary fluid (SSF) and 25 µL of CaCl_2_(H_2_O)_2_. The pH was adjusted to 7, and redistilled water was added to reach a total volume of 10 mL. After a 3-min OP, 2 mL of the sample was removed and used for TPC determination, while 2 mL of the SSF was reintroduced into the system. After OP_3_ (the index represents the total digestion time in minutes), the GP initiated by adding 8 mL of simulated gastric fluid (SGF) and 5 µL of CaCl_2_(H_2_O)_2_ to the system. The pH was adjusted to 3 with 1 M HCl. Redistilled water was added to a final volume of 20 mL, and this phase lasted 120 min. At specific time intervals (GP_63_, GP_123_), 2 mL of the sample was removed and an equal volume of SGF was added back, corresponding to the approach used in OP. Finally, the intestinal phase (IP) was initiated by adding 16 mL of simulated intestinal fluid (SIF) and 40 µL of CaCl_2_(H_2_O)_2_. The pH was adjusted to 7, and redistilled water was added to bring the total volume to 40 mL. This phase also lasted 120 min, and the sampling and recovery of the same amount of SIF at one time interval of the phase (IP_183_) was consistent with the previous OP and GP, while at IP_243_ only sampling was conducted.

### 4.10. Statistical Analysis

To test the significance level of the difference between the arithmetic means of the samples representing the population, a one-way analysis of variance (ANOVA) was performed using TIBCO Statistica software (TIBCO Software Inc., Palo Alto, CA, USA). After determining statistically significant differences, an additional post-hoc test (Duncan’s test for multiple ranges) was performed to determine specific populations that showed significant differences (*p* < 0.05) [68]. Samples belonging to the same population were marked with the same letter of the alphabet in the figures or tables.

## Figures and Tables

**Table 1:** Coatings used for encapsulation.

#	Coating	Encapsulation Feed		Crosslinking Solution		Immersion Solution	
1	SA	CSE sodium alginate	1.0% 3.0%	CaCl_2_	0.25 M	-	
2	SA + MDl	CSE sodium alginate maltodextrin (DE 4–7)	1.0% 3.0% 1.2%	CaCl_2_	0.25 M	-	
3	SA + MDh	CSE sodium alginate maltodextrin (DE 16.5–19.5)	1.0% 3.0% 1.2%	CaCl_2_	0.25 M	-	
4	SA + GT	CSE sodium alginate gum Tragacanth	1.0% 3.0% 0.15%	CaCl_2_	0.25 M	-	
5	SA + GA	CSE sodium alginate gum Arabic	1.0% 3.0% 1.6%	CaCl_2_	0.25 M	-	
6	SA + GEL	CSE sodium alginate gelatin	1.0% 3.0% 5.0%	CaCl_2_	0.25 M	-	
7	SA/1.5CH	CSE sodium alginate	1.0% 3.0%	CaCl_2_ chitosan	0.25 M 1.5%	-	
8	SA/1.0CH	CSE sodium alginate	1.0% 3.0%	CaCl_2_ chitosan	0.25 M 1.0%	-	
9	SA/0.5CH	CSE sodium alginate	1.0% 3.0%	CaCl_2_ chitosan	0.25 M 0.5%	-	
10	SA(1.5CH)	CSE sodium alginate	1.0% 3.0%	CaCl_2_	0.25 M	chitosan	1.5%
11	SA(1.0CH)	CSE sodium alginate	1.0% 3.0%	CaCl_2_	0.25 M	chitosan	1.0%
12	SA(0.5CH)	CSE sodium alginate	1.0% 3.0%	CaCl_2_	0.25 M	chitosan	0.5%

## Data Availability

The data presented in this study are openly available in article.

## Supplementary Materials

The following supporting information can be downloaded at: https://www.mdpi.com/article/10.3390/gels9110870/s1, Figure S1: SEM image of the grape pomace extract encapsulated in air-dried (AD), vacuum-dried (VD), and freeze-dried (FD) microbeads prepared using various coatings (SA–sodium alginate, and combinations of sodium alginate with maltodextrin (dextrose equivalent 4–7)–SA + MDl, maltodextrin (dextrose equivalent 16.5–19.5)–SA + MDh, gum Arabica–SA + GA, gelatin–SA + GEL; with chitosan (CH) dispersed in crosslinking solution at various concentrations: SA/1.5CH–1.5% (*w*/*v*), SA/1.0CH–1.0% (*w*/*v*), SA/0.5CH–0.5% (*w*/*v*); and with CH when alginate hydrogels were immersed in CH solution (image of freeze dried microbeads presented in manuscript) of various concentrations: SA(1.5CH)–1.5% (*w*/*v*), SA(1.0CH)–1.0% (*w*/*v*), SA(0.5CH)–0.5% (*w*/*v*); Figure S2. Cumulative release of total phenolic compounds (TPC) from air-dried, vacuum-dried, and freeze-dried microbeads (MB) containing grape pomace extract and coated with sodium alginate (SA) (OP-oral phase, GP-gastric phase, IP-intestinal phase, index number–duration of digestion in vitro); Figure S3. Cumulative release of total phenolic compounds (TPC) from air-dried, vacuum-dried, and freeze-dried microbeads (MB) containing grape pomace extract and coated with sodium alginate (SA) combined with (A) maltodextrin DE 4–7 (SA + MDl); (B) maltodextrin DE 16.5–19.5 (SA + MDh); (C) gum Tragacanth (SA + GT), (D) gum Arabica (SA + GA); (E) gelatin (SA + GEL) (OP-oral phase, GP-gastric phase, IP-intestinal phase, index number–duration of digestion in vitro); Figure S4: Cumulative release of total phenolic compounds (TPC) from air-dried, vacuum-dried, and freeze-dried microbeads (MB) containing grape pomace extract and coated with sodium alginate (SA) combined with chitosan dispersed in crosslinking solution in various concentrations: (A) 1.5% (SA/1.5CH); (B) 1.0% (SA/1.0CH); and (C) 0.5% (SA/0.5CH) (OP-oral phase, GP-gastric phase, IP-intestinal phase, index number–duration of digestion in vitro); Figure S5. Cumulative release of total phenolic compounds (TPC) from air-dried, vacuum-dried, and freeze-dried microbeads (MB) containing grape pomace extract and coated with sodium alginate (SA) and subsequently immersed in chitosan (CH) in various concentrations: (A) 1.5% (SA(1.5CH)); (B) 1.0% (SA(1.0CH)); and (C) 0.5% (SA/(0.5CH)), (OP-oral phase, GP-gastric phase, IP-intestinal phase, index number–duration of digestion in vitro). Table S1: Area of hydrogel microbeads and dried microbeads.; Table S2: Perimeter of hydrogel microbeads and dried microbeads; Table S3: Feret_MAX_ of hydrogel microbeads and dried microbeads; Table S4: Feret_MIN_ of hydrogel microbeads and dried microbeads; Table S5. Circularity of hydrogel microbeads and dried microbeads; Table S6. Roundness of hydrogel microbeads and dried microbeads; Table S7. Solidity of hydrogel microbeads and dried microbeads; Table S8. Hardness of hydrogel microbeads and dried microbeads.
