# Supplementary material for: The Release of Grape Pomace Phenolics from Alginate-Based Microbeads during Simulated Digestion In Vitro: The Influence of Coatings and Drying Method
Source: Gels. 2023 Nov 1;9(11):870. doi: 10.3390/gels9110870 (PMC10671312; doi:10.3390/gels9110870)
Supplement: Supplementary file 1 [file gels-09-00870-s001.zip › gels-2695038-supplementary.pdf]

Supplementary Materials

The Release of Grape Pomace Phenolics from Alginate-Based Microbeads during Simulated Digestion In Vitro: The Influence of Coatings and Drying Method

Table S1. Area of hydrogel microbeads and dried microbeads.

#	Coating	Hydrogels ^{A}	Microbeads		
			Air-dried ^{C}	Vacuum-dried ^{C}	Freeze-dried ^{B}
		**Area (mm^2)**			
1	**SA**	11.84 ± 0.77 ^{f}	1.91 ± 0.22 ^{e}	2.11 ± 0.24 ^{e}	4.50 ± 1.15 ^{f}
2	**SA+MDl**	13.25 ± 1.00 ^{e}	2.19 ± 0.32 ^{c}	2.68 ± 0.39 ^{b}	6.60 ± 0.95 ^{e}
3	**SA+MDh**	13.08 ± 0.98 ^{e}	2.04 ± 0.26 ^{de}	2.46 ± 0.30 ^{cd}	6.17 ± 1.12 ^{e}
4	**SA+GT**	13.31 ± 1.16 ^{e}	2.16 ± 0.31 ^{cd}	1.92 ± 0.20 ^{f}	6.35 ± 1.10 ^{e}
5	**SA+GA**	14.61 ± 0.90 ^{d}	2.26 ± 0.20 ^{bc}	2.34 ± 0.20 ^{d}	10.12 ± 0.78 ^{a}
6	**SA+GEL**	16.56 ± 1.06 ^{c}	2.87 ± 0.23 ^{a}	3.36 ± 0.43 ^{a}	10.26 ± 0.80 ^{a}
7	**SA/1.5CH**	19.32 ± 1.90 ^{a}	2.27 ± 0.46 ^{bc}	2.51 ± 0.48 ^{c}	10.54 ± 1.97 ^{a}
8	**SA/1.0CH**	17.46 ± 1.52 ^{b}	2.14 ± 0.24 ^{cd}	2.38 ± 0.32 ^{cd}	8.45 ± 1.39 ^{bc}
9	**SA/0.5CH**	17.78 ± 1.32 ^{b}	2.36 ± 0.35 ^{b}	2.33 ± 0.35 ^{d}	8.88 ± 1.17 ^{b}
10	**SA(1.5CH)**	14.66 ± 0.87 ^{d}	1.59 ± 0.18 ^{f}	1.89 ± 0.26 ^{f}	8.16 ± 1.47 ^{cd}
11	**SA(1.0CH)**	13.09 ± 0.82 ^{e}	1.43 ± 0.11 ^{g}	1.66 ± 0.17 ^{g}	6.22 ± 1.11 ^{e}
12	**SA(0.5CH)**	13.24 ± 0.66 ^{e}	1.56 ± 0.25 ^{fg}	1.57 ± 0.18 ^{g}	7.71 ± 1.43 ^{d}

SA – sodium alginate, combinations of sodium alginate: with maltodextrin (dextrose equivalent 4–7) – SA + MDl, maltodextrin (dextrose equivalent 16.5–19.5) – SA + MDh , gum Tragacanth – SA + GT , gum Arabica – SA + GA , gelatin – SA + GEL; with chitosan (CH) dispersed in crosslinking solution at various concentrations: SA/1.5CH – 1.5% (*w/v*), SA/1.0CH – 1.0% (*w/v*), SA/0.5CH – 0.5 % (*w/v*); and with CH when alginate hydrogels were immersed in CH solution of various concentrations: SA(1.5CH) – 1.5% (*w/v*), SA(1.0CH) – 1.0% (*w/v*), SA(0.5CH) – 0.5% (*w/v*)). Different lowercase letters in the same column (a, b, c, …) represent significant differences between the coatings used, while uppercase letters in the exponent (A, B, C) represent significant differences between the microbeads (hydrogel and dried microbeads) regardless of the coating used (ANOVA, Duncan's at $p < 0.05$).

Table S2. Perimeter of hydrogel microbeads and dried microbeads.

#	Coating	Hydrogels ^{A}	Microbeads		
			Air-dried ^{D}	Vacuum-dried ^{C}	Freeze-dried ^{B}
		**Perimeter (mm)**			
1	**SA**	13.21 ± 0.51 ^{h}	5.21 ± 0.30 ^{f}	5.57 ± 0.34 ^{g}	8.57 ± 0.84 ^{f}
2	**SA+MDl**	14.13 ± 0.78 ^{ef}	5.59 ± 0.40 ^{de}	6.58 ± 0.82 ^{c}	10.32 ± 0.88 ^{e}
3	**SA+MDh**	13.71 ± 0.54 ^{fg}	5.42 ± 0.36 ^{ef}	6.01 ± 0.39 ^{ef}	10.27 ± 1.35 ^{e}
4	**SA+GT**	14.03 ± 0.82 ^{fg}	5.82 ± 0.57 ^{cd}	5.20 ± 0.27 ^{hi}	10.17 ± 0.88 ^{e}
5	**SA+GA**	14.50 ± 0.46 ^{de}	5.70 ± 0.30 ^{cd}	5.92 ± 0.30 ^{f}	13.46 ± 1.08 ^{b}
6	**SA+GEL**	16.09 ± 1.15 ^{c}	6.56 ± 0.65 ^{a}	7.29 ± 0.45 ^{a}	13.45 ± 0.72 ^{b}
7	**SA/1.5CH**	17.91 ± 1.04 ^{a}	6.20 ± 0.55 ^{b}	6.85 ± 0.77 ^{b}	14.56 ± 1.94 ^{a}
8	**SA/1.0CH**	16.74 ± 1.25 ^{b}	5.87 ± 0.36 ^{c}	6.49 ± 0.55 ^{cd}	12.28 ± 1.30 ^{c}
9	**SA/0.5CH**	16.57 ± 0.97 ^{b}	6.42 ± 0.98 ^{ab}	6.25 ± 0.81 ^{de}	12.91 ± 1.53 ^{b}
10	**SA(1.5CH)**	14.61 ± 0.55 ^{d}	4.80 ± 0.28 ^{g}	5.28 ± 0.32 ^{h}	11.63 ± 1.14 ^{d}
11	**SA(1.0CH)**	13.65 ± 0.44 ^{g}	4.56 ± 0.23 ^{g}	4.98 ± 0.31 ^{ij}	10.20 ± 0.96 ^{e}
12	**SA(0.5CH)**	13.78 ± 0.69 ^{fg}	4.70 ± 0.34 ^{g}	4.49 ± 0.29 ^{j}	11.24 ± 1.05 ^{d}

SA – sodium alginate, combinations of sodium alginate: with maltodextrin (dextrose equivalent 4–7) – SA + MDl, maltodextrin (dextrose equivalent 16.5–19.5) – SA + MDh , gum Tragacanth – SA + GT , gum Arabica – SA + GA , gelatin – SA+ GEL; with chitosan (CH) dispersed in crosslinking solution at various concentrations: SA/1.5CH – 1.5% (*w/v*), SA/1.0CH – 1.0% (*w/v*), SA/0.5CH – 0.5% (*w/v*); and with CH when alginate hydrogels were immersed in CH solution of various concentrations: SA(1.5CH) – 1.5% (*w/v*), SA(1.0CH) – 1.0% (*w/v*), SA(0.5CH) – 0.5% (*w/v*)). Different lowercase letters in the same column (a, b, c, …) represent significant differences between the coatings used, while uppercase letters in the exponent (A, B, C, D) represent

significant differences between the microbeads (hydrogel and dried microbeads) regardless of the coating used (ANOVA, Duncan's at $p < 0.05$).

Table S3. $Feret_{MAX}$ of hydrogel microbeads and dried microbeads.

#	Coating	Hydrogels [A]	Microbeads		
			Air-dried [D]	Vacuum-dried [C]	Freeze-dried [B]
		**$Feret_{MAX}$ (mm)**			
1	**SA**	4.24 ± 0.19 [g]	1.72 ± 0.09 [f]	1.85 ± 0.14 [f]	2.84 ± 0.26 [e]
2	**SA+MDl**	4.46 ± 0.24 [ef]	1.82 ± 0.14 [de]	2.09 ± 0.15 [d]	3.30 ± 0.28 [d]
3	**SA+MDh**	4.34 ± 0.20 [fg]	1.78 ± 0.12 [ef]	1.97 ± 0.12 [e]	3.28 ± 0.35 [d]
4	**SA+GT**	4.41 ± 0.26 [fg]	1.93 ± 0.15 [c]	1.70 ± 0.09 [gh]	3.30 ± 0.28 [d]
5	**SA+GA**	4.62 ± 0.15 [e]	1.88 ± 0.09 [cd]	1.96 ± 0.11 [e]	4.12 ± 0.28 [b]
6	**SA+GEL**	4.99 ± 0.39 [d]	2.11 ± 0.13 [b]	2.40 ± 0.13 [b]	4.11 ± 0.33 [b]
7	**SA/1.5CH**	6.65 ± 0.59 [a]	2.35 ± 0.22 [a]	2.57 ± 0.34 [a]	5.06 ± 0.66 [a]
8	**SA/1.0CH**	5.94 ± 0.78 [b]	2.13 ± 0.20 [b]	2.36 ± 0.27 [b]	4.01 ± 0.47 [b]
9	**SA/0.5CH**	5.55 ± 0.58 [c]	2.19 ± 0.32 [b]	2.22 ± 0.37 [c]	4.22 ± 0.71 [b]
10	**SA(1.5CH)**	4.63 ± 0.18 [e]	1.61 ± 0.07 [g]	1.78 ± 0.13 [fg]	3.70 ± 0.33 [c]
11	**SA(1.0CH)**	4.37 ± 0.13 [fg]	1.51 ± 0.07 [h]	1.65 ± 0.10 [hi]	3.41 ± 0.26 [d]
12	**SA(0.5CH)**	4.37 ± 0.15 [fg]	1.56 ± 0.11 [gh]	1.59 ± 0.08 [i]	3.68 ± 0.35 [c]

SA – sodium alginate, combinations of sodium alginate: with maltodextrin (dextrose equivalent 4–7) – SA + MDl, maltodextrin (dextrose equivalent 16.5–19.5) – SA + MDh , gum Tragacanth – SA + GT , gum Arabica – SA + GA , gelatin – SA+ GEL; with chitosan (CH) dispersed in crosslinking solution at various concentrations: SA/1.5CH – 1.5% (*w*/*v*), SA/1.0CH – 1.0% (*w*/*v*), SA/0.5CH – 0.5% (*w*/*v*); and with CH when alginate hydrogels were immersed in CH solution of various concentrations: SA(1.5CH) – 1.5% (*w*/*v*), SA(1.0CH) – 1.0% (*w*/*v*), SA(0.5CH) – 0.5% (*w*/*v*)). Different lowercase letters in the same column (a, b, c, …) represent significant differences between the coatings used, while uppercase letters in the exponent (A, B, C, D) represent significant differences between the microbeads (hydrogel and dried microbeads) regardless of the coating used (ANOVA, Duncan's at $p < 0.05$).

Table S4. $Feret_{MIN}$ of hydrogel microbeads and dried microbeads.

#	Coating	Hydrogels [A]	Microbeads		
			Air-dried [D]	Vacuum-dried [C]	Freeze-dried [B]
		**$Feret_{MIN}$ (mm)**			
1	**SA**	3.65 ± 0.18 [e]	1.48 ± 0.13 [d]	1.54 ± 0.12 [d]	2.17 ± 0.30 [e]
2	**SA+MDl**	3.88 ± 0.24 [d]	1.58 ± 0.13 [bc]	1.72 ± 0.17 [b]	2.67 ± 0.23 [cd]
3	**SA+MDh**	3.91 ± 0.22 [d]	1.52 ± 0.11 [cd]	1.67 ± 0.13 [bc]	2.57 ± 0.28 [d]
4	**SA+GT**	3.91 ± 0.21 [d]	1.52 ± 0.15 [cd]	1.48 ± 0.08 [de]	2.61 ± 0.28 [d]
5	**SA+GA**	4.11 ± 0.19 [b]	1.61 ± 0.09 [b]	1.63 ± 0.10 [c]	3.34 ± 0.22 [a]
6	**SA+GEL**	4.39 ± 0.16 [a]	1.81 ± 0.10 [a]	1.89 ± 0.22 [a]	3.38 ± 0.19 [a]
7	**SA/1.5CH**	3.89 ± 0.33 [d]	1.31 ± 0.20 [f]	1.34 ± 0.19 [f]	2.99 ± 0.39 [b]
8	**SA/1.0CH**	4.04 ± 0.25 [bc]	1.41 ± 0.16 [e]	1.43 ± 0.17 [e]	2.98 ± 0.30 [b]
9	**SA/0.5CH**	4.32 ± 0.29 [a]	1.54 ± 0.16 [bcd]	1.49 ± 0.17 [de]	2.98 ± 0.30 [b]
10	**SA(1.5CH)**	4.13 ± 0.22 [b]	1.33 ± 0.09 [f]	1.44 ± 0.13 [e]	2.99 ± 0.29 [b]
11	**SA(1.0CH)**	3.88 ± 0.17 [d]	1.27 ± 0.07 [f]	1.36 ± 0.10 [f]	2.52 ± 0.34 [d]
12	**SA(0.5CH)**	3.93 ± 0.16 [cd]	1.34 ± 0.11 [f]	1.32 ± 0.11 [f]	2.82 ± 0.34 [c]

SA – sodium alginate, combinations of sodium alginate: with maltodextrin (dextrose equivalent 4–7) – SA + MDl, maltodextrin (dextrose equivalent 16.5–19.5) – SA + MDh , gum Tragacanth – SA + GT , gum Arabica – SA + GA , gelatin – SA+ GEL; with chitosan (CH) dispersed in crosslinking solution at various concentrations: SA/1.5CH – 1.5% (*w*/*v*), SA/1.0CH – 1.0% (*w*/*v*), SA/0.5CH – 0.5% (*w*/*v*); and with CH when alginate hydrogels were immersed in CH solution of various concentrations: SA(1.5CH) – 1.5% (*w*/*v*), SA(1.0CH) – 1.0% (*w*/*v*), SA(0.5CH) – 0.5% (*w*/*v*)). Different lowercase letters in the same column (a, b, c,

…) represent significant differences between the coatings used, while uppercase letters in the exponent (A, B, C, D) represent significant differences between the microbeads (hydrogel and dried microbeads) regardless of the coating used (ANOVA, Duncan's at $p < 0.05$).

Table S5. Circularity of hydrogel microbeads and dried microbeads.

#	Coating	Hydrogels ^{A}	Microbeads		
			Air-dried ^{A}	Vacuum-dried ^{B}	Freeze-dried ^{C}
		Circularity (-)			
1	**SA**	0.85 ± 0.04 ^{bc}	0.88 ± 0.04 ^{a}	0.85 ± 0.05 ^{ab}	0.76 ± 0.07 ^{ab}
2	**SA+MDl**	0.84 ± 0.06 ^{cd}	0.88 ± 0.04 ^{a}	0.79 ± 0.11 ^{c}	0.78 ± 0.06 ^{a}
3	**SA+MDh**	0.87 ± 0.02 ^{ab}	0.87 ± 0.05 ^{a}	0.86 ± 0.04 ^{ab}	0.74 ± 0.12 ^{abc}
4	**SA+GT**	0.85 ± 0.05 ^{bc}	0.81 ± 0.07 ^{b}	0.89 ± 0.02 ^{a}	0.77 ± 0.07 ^{a}
5	**SA+GA**	0.87 ± 0.02 ^{ab}	0.87 ± 0.04 ^{a}	0.84 ± 0.05 ^{b}	0.71 ± 0.08 ^{cd}
6	**SA+GEL**	0.81 ± 0.07 ^{ef}	0.85 ± 0.10 ^{a}	0.79 ± 0.06 ^{c}	0.72 ± 0.06 ^{bcd}
7	**SA/1.5CH**	0.76 ± 0.04 ^{g}	0.74 ± 0.07 ^{c}	0.68 ± 0.09 ^{e}	0.63 ± 0.11 ^{e}
8	**SA/1.0CH**	0.79 ± 0.07 ^{f}	0.78 ± 0.06 ^{b}	0.71 ± 0.10 ^{d}	0.71 ± 0.08 ^{cd}
9	**SA/0.5CH**	0.82 ± 0.06 ^{de}	0.74 ± 0.12 ^{c}	0.76 ± 0.10 ^{c}	0.68 ± 0.10 ^{d}
10	**SA(1.5CH)**	0.86 ± 0.03 ^{ab}	0.87 ± 0.05 ^{a}	0.85 ± 0.05 ^{ab}	0.76 ± 0.06 ^{ab}
11	**SA(1.0CH)**	0.88 ± 0.01 ^{a}	0.87 ± 0.06 ^{a}	0.85 ± 0.06 ^{b}	0.75 ± 0.07 ^{abc}
12	**SA(0.5CH)**	0.88 ± 0.05 ^{ab}	0.89 ± 0.03 ^{a}	0.86 ± 0.03 ^{ab}	0.76 ± 0.07 ^{a}

SA – sodium alginate, combinations of sodium alginate: with maltodextrin (dextrose equivalent 4–7) – SA + MDl, maltodextrin (dextrose equivalent 16.5–19.5) – SA + MDh , gum Tragacanth – SA + GT , gum Arabica – SA + GA , gelatin – SA+ GEL; with chitosan (CH) dispersed in crosslinking solution at various concentrations: SA/1.5CH – 1.5% (*w/v*), SA/1.0CH – 1.0% (*w/v*), SA/0.5CH – 0.5% (*w/v*); and with CH when alginate hydrogels were immersed in CH solution of various concentrations: SA(1.5CH) – 1.5% (*w/v*), SA(1.0CH) – 1.0% (*w/v*), SA(0.5CH) – 0.5% (*w/v*)). Different lowercase letters in the same column (a, b, c, …) represent significant differences between the coatings used, while uppercase letters in the exponent (A, B, C, D) represent significant differences between the microbeads (hydrogel and dried microbeads) regardless of the coating used (ANOVA, Duncan's at $p < 0.05$).

Table S6. Roundness of hydrogel microbeads and dried microbeads.

#	Coating	Hydrogels ^{A}	Microbeads		
			Air-dried ^{B}	Vacuum-dried ^{C}	Freeze-dried ^{C}
		Roundness (-)			
1	**SA**	0.89 ± 0.06 ^{a}	0.88 ± 0.08 ^{a}	0.86 ± 0.08 ^{ab}	0.79 ± 0.09 ^{bcd}
2	**SA+MDl**	0.89 ± 0.05 ^{a}	0.89 ± 0.07 ^{a}	0.83 ± 0.08 ^{bc}	0.84 ± 0.07 ^{ab}
3	**SA+MDh**	0.91 ± 0.05 ^{a}	0.87 ± 0.06 ^{a}	0.87 ± 0.07 ^{ab}	0.80 ± 0.09 ^{abc}
4	**SA+GT**	0.90 ± 0.06 ^{a}	0.80 ± 0.08 ^{b}	0.89 ± 0.05 ^{a}	0.81 ± 0.08 ^{abc}
5	**SA+GA**	0.91 ± 0.05 ^{a}	0.88 ± 0.05 ^{a}	0.86 ± 0.07 ^{ab}	0.83 ± 0.09 ^{ab}
6	**SA+GEL**	0.90 ± 0.06 ^{a}	0.87 ± 0.06 ^{a}	0.80 ± 0.11 ^{c}	0.85 ± 0.08 ^{a}
7	**SA/1.5CH**	0.59 ± 0.09 ^{d}	0.56 ± 0.09 ^{e}	0.52 ± 0.12 ^{f}	0.60 ± 0.11 ^{e}
8	**SA/1.0CH**	0.70 ± 0.11 ^{c}	0.68 ± 0.12 ^{d}	0.62 ± 0.12 ^{e}	0.79 ± 0.12 ^{bcd}
9	**SA/0.5CH**	0.81 ± 0.11 ^{b}	0.74 ± 0.13 ^{c}	0.70 ± 0.16 ^{d}	0.74 ± 0.14 ^{d}
10	**SA(1.5CH)**	0.92 ± 0.05 ^{a}	0.85 ± 0.06 ^{a}	0.83 ± 0.09 ^{bc}	0.83 ± 0.07 ^{ab}
11	**SA(1.0CH)**	0.90 ± 0.04 ^{a}	0.86 ± 0.07 ^{a}	0.84 ± 0.09 ^{abc}	0.76 ± 0.12 ^{cd}
12	**SA(0.5CH)**	0.91 ± 0.04 ^{a}	0.88 ± 0.06 ^{a}	0.85 ± 0.08 ^{abc}	0.78 ± 0.10 ^{bcd}

SA – sodium alginate, combinations of sodium alginate: with maltodextrin (dextrose equivalent 4–7) – SA + MDl, maltodextrin (dextrose equivalent 16.5–19.5) – SA + MDh , gum Tragacanth – SA + GT , gum Arabica – SA + GA , gelatin – SA + GEL; with chitosan (CH) dispersed in crosslinking solution at various concentrations: SA/1.5CH – 1.5% (*w/v*), SA/1.0CH – 1.0% (*w/v*), SA/0.5CH – 0.5% (*w/v*); and with CH when alginate hydrogels were immersed in CH solution of various concentrations:

SA(1.5CH) – 1.5% (*w/v*), SA(1.0CH) – 1.0% (*w/v*), SA(0.5CH) – 0.5% (*w/v*)). Different lowercase letters in the same column (a, b, c, …) represent significant differences between the coatings used, while uppercase letters in the exponent (A, B, C, D) represent significant differences between the microbeads (hydrogel and dried microbeads) regardless of the coating used (ANOVA, Duncan's at $p < 0.05$).

Table S7. Solidity of hydrogel microbeads and dried microbeads.

#	Coating	Hydrogels ^{A}	Microbeads		
			Air-dried ^{B}	Vacuum-dried ^{C}	Freeze-dried ^{C}
		Solidity (-)			
1	**SA**	0.98 ± 0.00 ^{ab}	0.96 ± 0.01 ^{a}	0.96 ± 0.01 ^{abc}	0.94 ± 0.03 ^{bc}
2	**SA+MDl**	0.98 ± 0.01 ^{bc}	0.97 ± 0.01 ^{a}	0.95 ± 0.03 ^{cd}	0.96 ± 0.01 ^{a}
3	**SA+MDh**	0.98 ± 0.00 ^{a}	0.96 ± 0.02 ^{ab}	0.96 ± 0.01 ^{ab}	0.95 ± 0.03 ^{ab}
4	**SA+GT**	0.98 ± 0.01 ^{a}	0.95 ± 0.02 ^{bc}	0.97 ± 0.01 ^{a}	0.96 ± 0.02 ^{a}
5	**SA+GA**	0.98 ± 0.01 ^{a}	0.97 ± 0.01 ^{a}	0.95 ± 0.01 ^{bc}	0.96 ± 0.01 ^{a}
6	**SA+GEL**	0.97 ± 0.02 ^{c}	0.96 ± 0.02 ^{a}	0.95 ± 0.02 ^{bc}	0.96 ± 0.01 ^{a}
7	**SA/1.5CH**	0.97 ± 0.01 ^{c}	0.94 ± 0.02 ^{c}	0.93 ± 0.03 ^{e}	0.93 ± 0.03 ^{c}
8	**SA/1.0CH**	0.97 ± 0.02 ^{c}	0.95 ± 0.01 ^{c}	0.92 ± 0.03 ^{e}	0.94 ± 0.02 ^{bc}
9	**SA/0.5CH**	0.98 ± 0.01 ^{bc}	0.93 ± 0.04 ^{d}	0.94 ± 0.03 ^{d}	0.94 ± 0.03 ^{bc}
10	**SA(1.5CH)**	0.98 ± 0.01 ^{a}	0.96 ± 0.02 ^{ab}	0.96 ± 0.02 ^{abc}	0.96 ± 0.01 ^{a}
11	**SA(1.0CH)**	0.98 ± 0.00 ^{a}	0.96 ± 0.01 ^{ab}	0.96 ± 0.01 ^{abc}	0.95 ± 0.01 ^{ab}
12	**SA(0.5CH)**	0.98 ± 0.01 ^{a}	0.96 ± 0.01 ^{ab}	0.96 ± 0.01 ^{abc}	0.96 ± 0.01 ^{a}

SA – sodium alginate, combinations of sodium alginate: with maltodextrin (dextrose equivalent 4–7) – SA + MDl, maltodextrin (dextrose equivalent 16.5–19.5) – SA + MDh , gum Tragacanth – SA + GT , gum Arabica – SA + GA , gelatin – SA + GEL; with chitosan (CH) dispersed in crosslinking solution at various concentrations: SA/1.5CH – 1.5% (*w/v*), SA/1.0CH – 1.0% (*w/v*), SA/0.5CH – 0.5% (*w/v*); and with CH when alginate hydrogels were immersed in CH solution of various concentrations: SA(1.5CH) – 1.5% (*w/v*), SA(1.0CH) – 1.0% (*w/v*), SA(0.5CH) – 0.5% (*w/v*)). Different lowercase letters in the same column (a, b, c, …) represent significant differences between the coatings used, while uppercase letters in the exponent (A, B, C, D) represent significant differences between the microbeads (hydrogel and dried microbeads) regardless of the coating used (ANOVA, Duncan's at $p < 0.05$).

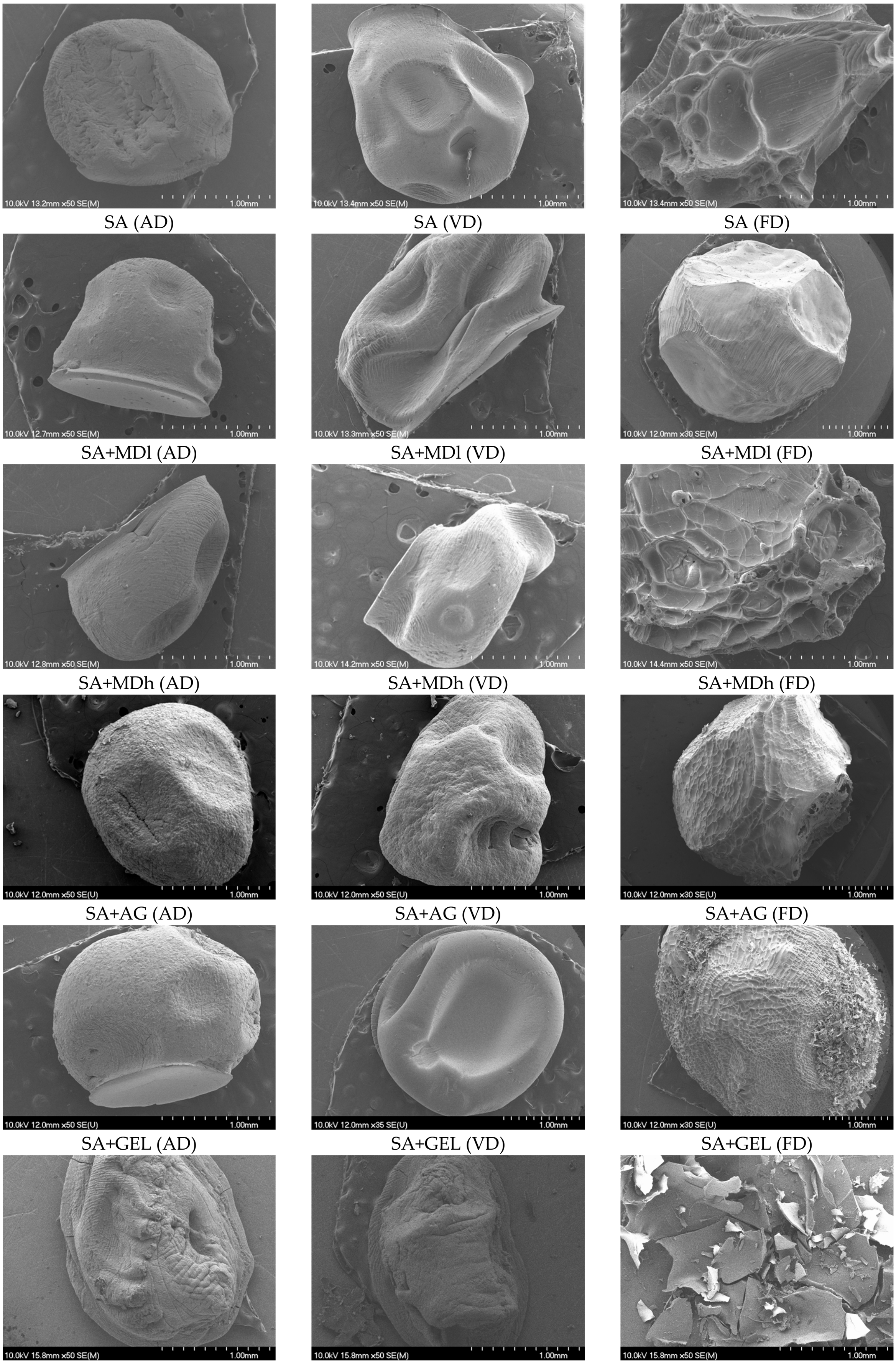
SA (AD)
SA (VD)
SA (FD)
SA+MDl (AD)
SA+MDl (VD)
SA+MDl (FD)
SA+MDh (AD)
SA+MDh (VD)
SA+MDh (FD)
SA+AG (AD)
SA+AG (VD)
SA+AG (FD)
SA+GEL (AD)
SA+GEL (VD)
SA+GEL (FD)
SA/1.0CH (AD)
SA/1.0CH (VD)
SA/1.0CH (FD)

Figure S1.

Continuing **Figure S1.**

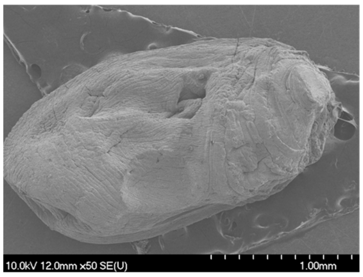

SA/0.5CH (AD)

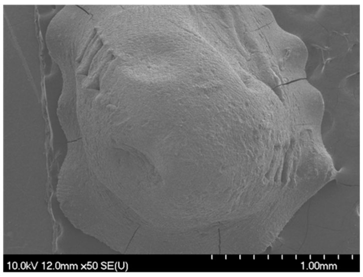

SA/0.5CH (VD)

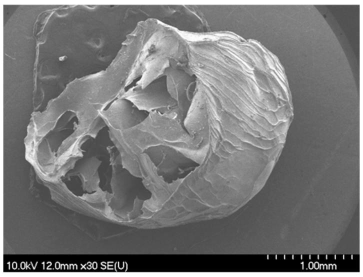

SA/0.5CH (FD)

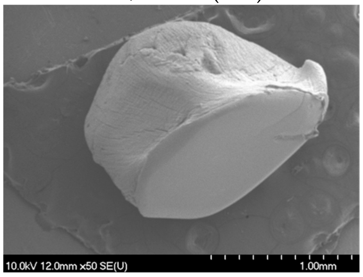

SA/(1.5CH) (AD)

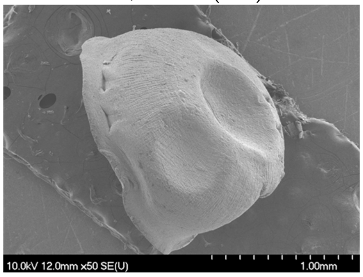

SA/(1.5CH) (VD)

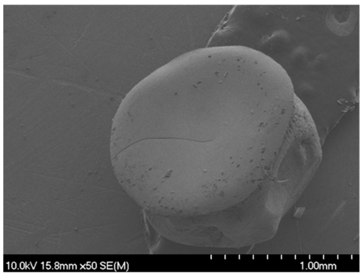

SA/(1.0CH) (AD)

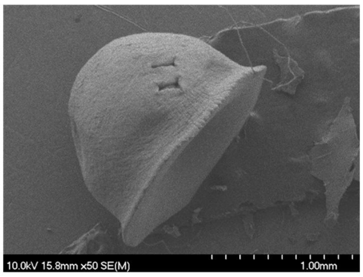

SA/(1.0CH) (VD)

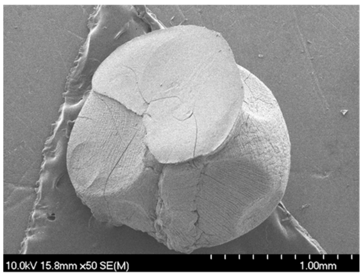

SA/(0.5CH) (AD)

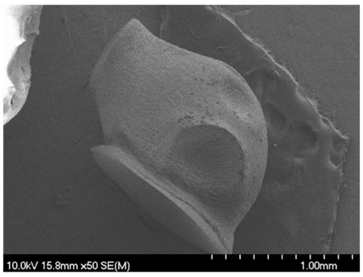

SA/(0.5CH) (VD)

Figure S1. SEM image of the grape pomace extract encapsulated in air-dried (AD), vacuum-dried (VD), and freeze-dried (FD) microbeads prepared using various coatings (SA – sodium alginate, and combinations of sodium alginate with maltodextrin (dextrose equivalent 4–7) – SA + MDl, maltodextrin (dextrose equivalent 16.5–19.5) – SA + MDh , gum Arabica – SA + GA , gelatin – SA + GEL; with chitosan (CH) dispersed in crosslinking solution at various concentrations: SA/1.5CH – 1.5% (*w*/*v*), SA/1.0CH – 1.0% (*w*/*v*), SA/0.5CH – 0.5% (*w*/*v*); and with CH when alginate hydrogels were immersed in CH solution (image of freeze dried microbeads presented in manuscript) of various concentrations: SA(1.5CH) – 1.5% (*w*/*v*), SA(1.0CH) – 1.0% (*w*/*v*), SA(0.5CH) – 0.5% (*w*/*v*)).

Table S8. Hardness of hydrogel microbeads and dried microbeads.

#	Coating	Hydrogels ^{C}	Microbeads		
			Air-dried ^{A}	Vacuum-dried ^{B}	Freeze-dried ^{C}
		Hardness (N)			
1	**SA**	0.40 ± 0.07 ^{abc}	31.53 ± 2.01 ^{ab}	30.76 ± 4.55 ^{ab}	3.40 ± 3.52 ^{a}
2	**SA+MDl**	0.31 ± 0.09 ^{de}	33.35 ± 6.72 ^{ab}	17.54 ± 3.10 ^{d}	2.02 ± 1.36 ^{ab}
3	**SA+MDh**	0.44 ± 0.16 ^{a}	35.03 ± 4.35 ^{ab}	27.90 ± 4.97 ^{abc}	0.93 ± 0.49 ^{b}
4	**SA+GT**	0.35 ± 0.09 ^{bcde}	31.78 ± 6.61 ^{ab}	20.61 ± 5.74 ^{d}	0.78 ± 0.29 ^{b}
5	**SA+GA**	0.32 ± 0.03 ^{cde}	28.58 ± 4.85 ^{b}	28.00 ± 4.02 ^{abc}	1.22 ± 0.43 ^{b}
6	**SA+GEL**	0.39 ± 0.10 ^{abcd}	33.99 ± 4.28 ^{ab}	17.88 ± 4.52 ^{d}	0.98 ± 0.66 ^{b}
7	**SA/1.5CH**	0.36 ± 0.07 ^{abcde}	37.91 ± 3.51 ^{a}	17.72 ± 7.31 ^{d}	0.49 ± 0.17 ^{b}
8	**SA/1.0CH**	0.30 ± 0.06 ^{e}	28.12 ± 10.61 ^{b}	10.83 ± 4.04 ^{e}	0.46 ± 0.28 ^{b}
9	**SA/0.5CH**	0.39 ± 0.08 ^{abcd}	27.50 ± 8.17 ^{b}	10.27 ± 6.00 ^{e}	0.48 ± 0.35 ^{b}
10	**SA(1.5CH)**	0.29 ± 0.07 ^{e}	38.51 ± 0.99 ^{a}	22.71 ± 3.45 ^{cd}	0.38 ± 0.10 ^{b}
11	**SA(1.0CH)**	0.34 ± 0.08 ^{bcde}	35.40 ± 9.05 ^{ab}	24.37 ± 6.37 ^{bcd}	1.06 ± 0.75 ^{b}
12	**SA(0.5CH)**	0.41 ± 0.06 ^{ab}	34.64 ± 6.38 ^{ab}	32.88 ± 5.32 ^{a}	0.71 ± 0.18 ^{b}

SA – sodium alginate, combinations of sodium alginate: with maltodextrin (dextrose equivalent 4–7) – SA + MDl, maltodextrin (dextrose equivalent 16.5–19.5) – SA + MDh , gum Tragacanth – SA + GT , gum Arabica – SA + GA , gelatin – SA + GEL; with chitosan (CH) dispersed in crosslinking solution at various concentrations: SA/1.5CH – 1.5% (*w/v*), SA/1.0CH – 1.0% (*w/v*), SA/0.5CH – 0.5% (*w/v*); and with CH when alginate hydrogels were immersed in CH solution of various concentrations: SA(1.5CH) – 1.5% (*w/v*), SA(1.0CH) – 1.0% (*w/v*), SA(0.5CH) – 0.5% (*w/v*)). Different lowercase letters in the same column (a, b, c, …) represent significant differences between the coatings used, while uppercase letters in the exponent (A, B, C, D) represent significant differences between the microbeads (hydrogel and dried microbeads) regardless of the coating used (ANOVA, Duncan's at $p < 0.05$).

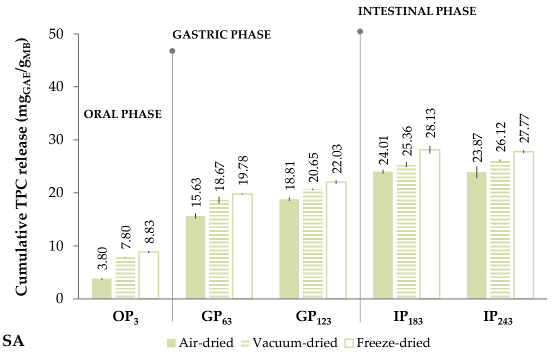

Figure S2. Cumulative release of total phenolic compounds (TPC) from air-dried, vacuum-dried, and freeze-dried microbeads (MB) containing grape pomace extract and coated with sodium alginate (SA) (OP-oral phase, GP-gastric phase, IP-intestinal phase, index number – duration of digestion in vitro).

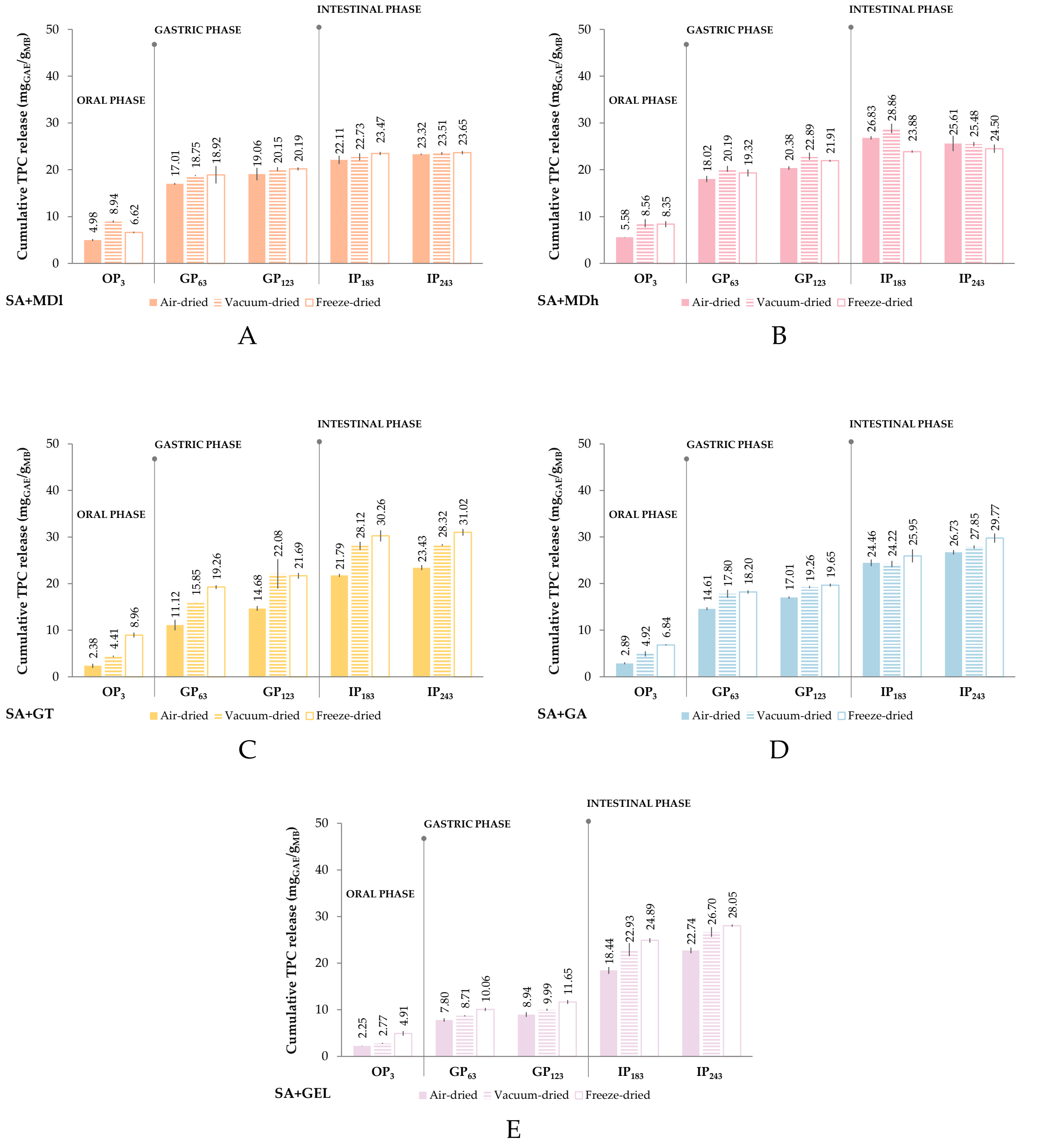

Figure S3. Cumulative release of total phenolic compounds (TPC) from air-dried, vacuum-dried, and freeze-dried microbeads (MB) containing grape pomace extract and coated with sodium alginate (SA) combined with (A) maltodextrin DE 4–7 (SA+ MDl); (B) maltodextrin DE 16.5 –19.5 (SA + MDh); (C) gum Tragacanth (SA + GT), (D) gum Arabica (SA + GA); (E) gelatin (SA + GEL) (OP-oral phase, GP-gastric phase, IP-intestinal phase, index number – duration of digestion in vitro).

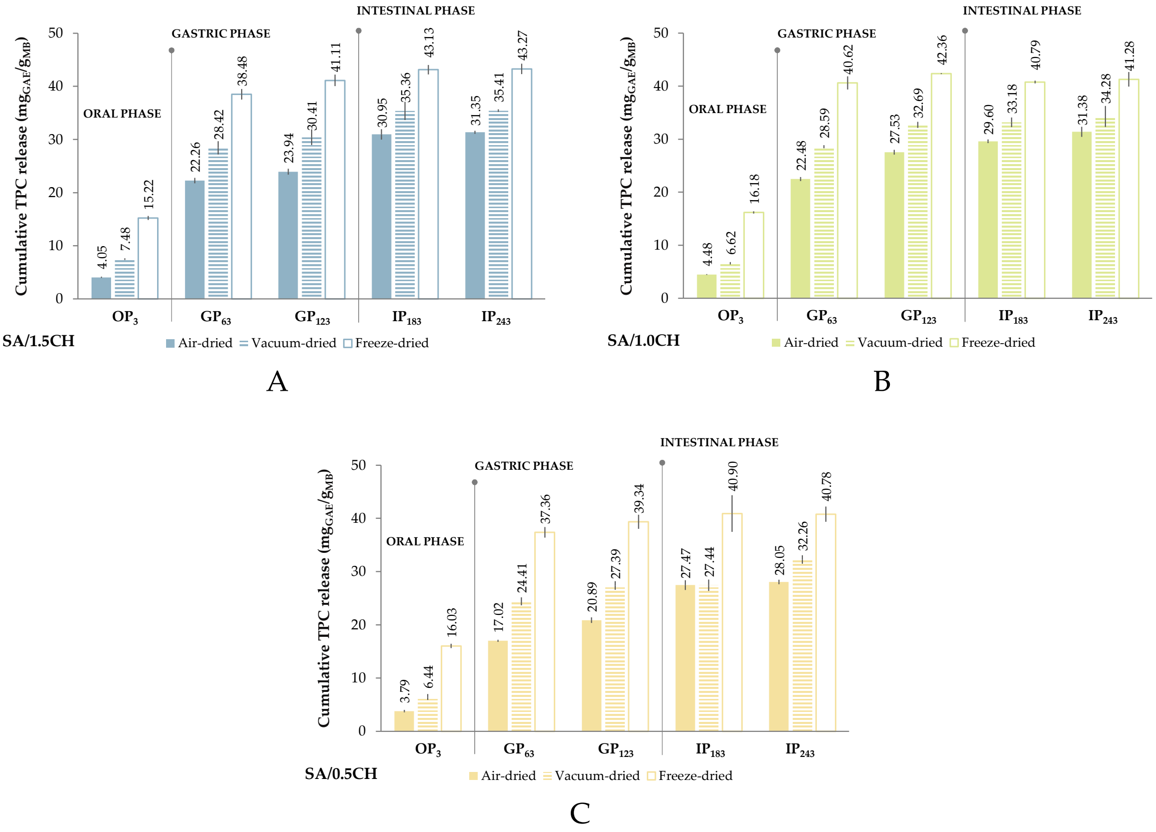

Figure S4. Cumulative release of total phenolic compounds (TPC) from air-dried, vacuum-dried, and freeze-dried microbeads (MB) containing grape pomace extract and coated with sodium alginate (SA) combined with chitosan dispersed in crosslinking solution in various concentrations: (A) 1.5% (SA/1.5CH); (B) 1.0% (SA/1.0CH); and (C) 0.5% (SA/0.5CH) (OP-oral phase, GP-gastric phase, IP-intestinal phase, index number – duration of digestion in vitro).

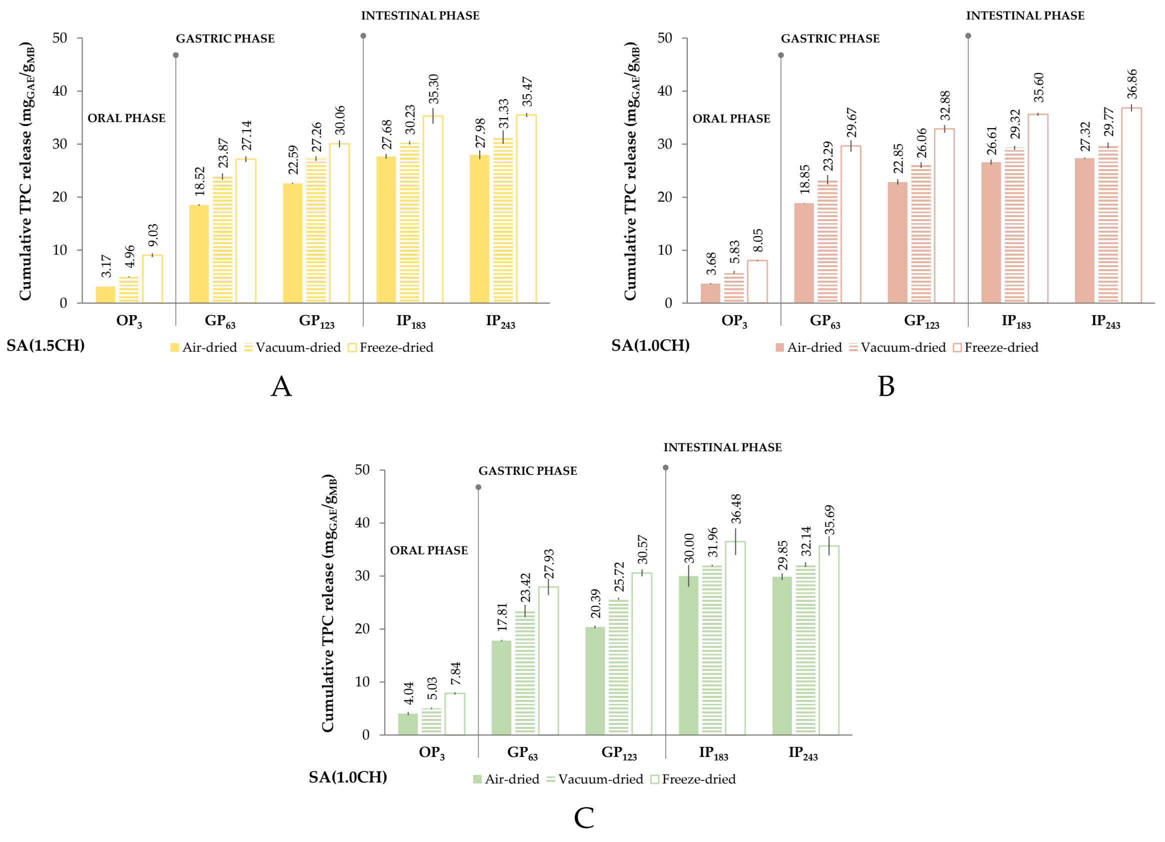

Figure S5. Cumulative release of total phenolic compounds (TPC) from air-dried, vacuum-dried, and freeze-dried microbeads (MB) containing grape pomace extract and coated with sodium alginate (SA) and subsequently immersed in chitosan (CH) in various concentrations: (A) 1.5% (SA(1.5CH)); (B) 1.0% (SA(1.0CH)); and (C) 0.5% (SA/(0.5CH)), (OP-oral phase, GP-gastric phase, IP-intestinal phase, index number – duration of digestion in vitro).
